# Developing an approach to assessing the political feasibility of global collective action and an international agreement on antimicrobial resistance

**DOI:** 10.1186/s41256-016-0020-9

**Published:** 2016-12-13

**Authors:** Susan Rogers Van Katwyk, Marie Évelyne Danik, Ioana Pantis, Rachel Smith, John-Arne Røttingen, Steven J. Hoffman

**Affiliations:** 1 Global Strategy Lab, Centre for Health Law, Policy & Ethics, Faculty of Law, University of Ottawa, Ottawa, Canada; 2 School of Epidemiology, Public Health, and Preventive Medicine, University of Ottawa, Ottawa, Canada; 3 Division of Infection Control & Environmental Health, Norwegian Institute of Public Health, Oslo, Norway; 4 Department of Health and Society, University of Oslo, Oslo, Norway; 5 Department of Clinical Epidemiology & Biostatistics and McMaster Health Forum, McMaster University, Hamilton, ON Canada; 6 Department of Global Health & Population, Harvard T.H. Chan School of Public Health, Harvard University, Boston, MA USA

**Keywords:** AMR, Antibiotic Resistance, Global Health, Political Feasibility, International Affairs

## Abstract

**Background:**

Antimicrobial resistance (AMR) is a global issue. International trade, travel, agricultural practices, and environmental contamination all make it possible for resistant microbes to cross national borders. Global collective action is needed in the form of an international agreement or other mechanism that brings states together at the negotiation table and commits them to adopt or implement policies to limit the spread of resistant microorganisms. This article describes an approach to assessing whether political and stakeholder interests can align to commit to tackling AMR.

**Methods:**

Two dimensions affecting political feasibility were selected and compared across 82 countries: 1) states’ global influence and 2) self-interest in addressing AMR. World Bank GDP ranking was used as a proxy for global influence, while human antibiotic consumption (10-year percent change) was used as a proxy for self-interest in addressing AMR. We used these data to outline a typology of four country archetypes, and discuss how these archetypes can be used to understand whether a proposed agreement may have sufficient support to be politically feasible.

**Results:**

Four types of countries exist within our proposed typology: 1) wealthy countries who have the expertise and financial resources to push for global collective action on AMR, 2) wealthy countries who need to act on AMR, 3) countries who require external assistance to act on AMR, and 4) neutral countries who may support action where applicable. Any international agreement will require substantial support from countries of the first type to lead global action, and from countries of the second type who have large increasing antimicrobial consumption levels. A large number of barriers exist that could derail efforts towards global collective action on AMR; issues of capacity, infrastructure, regulation, and stakeholder interests will need to be addressed in coordination with other actors to achieve an agreement on AMR.

**Conclusions:**

Achieving a global agreement on access, conservation, and innovation – the three pillars of AMR – will not be easy. However, smaller core groups of interested Initiator and Pivotal Countries could develop policy and resolve many issues. If highly influential countries take the lead, agreements could then be scaled up to achieve global action.

**Electronic supplementary material:**

The online version of this article (doi:10.1186/s41256-016-0020-9) contains supplementary material, which is available to authorized users.

## Background

Antimicrobial resistance (AMR) occurs when microorganisms such as bacteria, viruses, fungi, and parasites evolve to become resistant to antimicrobial medicines [1, 2]. International travel, trade, agricultural practices, and environmental contamination make it easy for resistant microbes to cross national borders [3]. Today, approximately 700,000 people worldwide die each year from AMR infections [4].

Antibiotic resistance has consequences for health, economics and society. One estimate predicts that AMR will cause 10 million annual deaths and $100 trillion in cumulative gross domestic product (GDP) loss by the year 2050 [4]. Surveillance data suggests that there are very high rates of antimicrobial resistance throughout all WHO regions [5]. Many organisms have developed resistance, and the effects are widely felt. For example, bacteria, such as methicillin resistance *staphylococcus aureus* and *Klebsiella pneumonia* are associated with high levels of resistance in hospitals, while the sexually transmitted infection *Neisseria gonorrhoea* is associated with resistance in the community. A key concern for many countries will be the rise of resistance to drugs for Tuberculosis, Malaria and HIV [5]. Thus, AMR is a global issue that requires swift global collective action to prevent a post-antimicrobial era [1, 3]. A comprehensive solution to AMR must address three interrelated concerns at the global level: 1) access; 2) conservation; and 3) innovation [6]. Inappropriate and excessive use of antimicrobials accelerates the development of AMR [7]. A lack of innovation means that stores of effective antimicrobials are depleting [1]. Meanwhile, millions of people die each year because they cannot access effective drugs for antimicrobial-susceptible infections [8].

Ultimately, achieving global collective action through an international agreement or other mechanism requires states to come together at the negotiation table and commit to adopting and implementing decided policies [9]. Momentum for international action has been building on this front since the publication of the WHO Global Strategy for Containment of Antimicrobial Resistance in 2001 [10]. Antimicrobial resistance has been discussed several times at the World Health Assembly [9, 11–13] in the intervening years and in 2015 the WHO published a Global Action Plan on Antimicrobial Resistance [14]. Academics have called for global action on antimicrobial resistance many times in the recent years [6, 7, 15–18], and several financing initiatives have been launched to improve innovation of new antimicrobials (see Ardal 2016 for a summary [19].) Most recently, antimicrobial resistance was discussed at a High Level Meeting of the United Nations General Assembly in September 2016 [20].

Economic and budgetary realities, health systems capacities and resources, and competing national priorities can either constrain or empower governments to act on global issues such as AMR [21]. While an international agreement could initiate the necessary global collective action on AMR [6, 17], states are unlikely to support or implement such an agreement unless its provisions benefit domestic and stakeholder interests as well as international priorities [22, 23]. To be politically feasible, states and stakeholders must perceive that the benefits of any agreement outweigh the costs and potential harms.

This article describes an approach we developed for assessing the political feasibility of achieving global collective action and bringing states together to craft an international agreement on AMR. We define political feasibility in this context as the likelihood that political and stakeholder interests can align to allow nations to agree to and implement policies addressing antimicrobial access, conservation, and innovation. The described approach allows interested parties to understand whether a proposed international agreement may have sufficient support from key actors to be politically feasible.

## Methods

To analyze political feasibility, we must predict how a country will act in the face of different proposed international agreements and in accordance with its internal political and economic considerations. To this end we first created a typology that describes four archetypes of state actors and allows us to hypothesize whether an international agreement might be feasible based on the participation of interested states. To complement this analysis, we identified key barriers to early stage policymaking that might derail a global agreement on AMR. A more detailed description of methods and results is available in the Full Report/Additional file 1.

### Comparative political analysis

Two dimensions affecting political feasibility were selected and compared across 82 countries: 1) states’ global influence and 2) self-interest in addressing AMR. Since these dimensions are difficult to measure directly, proxy measures were selected that balanced the need to maximize correlation with the primary construct and availability of data for as many countries as possible.

GDP ranking was chosen to proxy for global influence, as economic power is often the key determinant of global importance. States with financial resources are able to purchase favour, finance global initiatives, and attract support. The World Bank makes available annual GDP data for 214 economies [24].

Changes in human antibiotic consumption was chosen to proxy for self-interest in addressing AMR. Ideally we would have used national AMR rates, but unfortunately there is very little comparable national data available, particularly for Low and Middle Income Countries (LMIC). Instead, we used 10-year percent change in human antibiotic use. This is a dynamic measure that allows us to account for trends in consumption and behaviour changes, while limiting the potential to be misled by short-term health and market shocks. Such a 10-year percent change in antimicrobial consumption gives a long-term overview of antimicrobial importance at the national level. We interpreted this measure as an indicator of action or inaction on AMR, which we assume is correlated with states’ self-interest in addressing AMR. Whether the country had high or low consumption rates in 2000, a large proportional increase in consumption signals a need to re-examine national priorities, while a decrease suggests successful stewardship efforts.

Data was available to plot GDP ranking against 10-year percent change in human antibiotic consumption for 82 countries.

### Data sources and data treatment

GDP data was obtained from the World Bank’s 2014 GDP rankings [24]. Data for 10-year percent change in human antibiotic consumption was obtained from the Center for Disease Dynamics, Economics & Policy (CDDEP) and was collected for the period from 2000 to 2010. This CDDEP data comprises information collected in the IMS Health MIDAS database, which uses antibiotics sold in retail and hospital pharmacies to estimate antibiotic consumption. Details on the methods of CDDEP can be found elsewhere [25]. Individual data was available for 66 countries, while data for 6 countries in Central America and 10 countries in French West Africa were reported as a group using regional estimates of antibiotic consumption. We chose to include these countries individually on the chart. Each country was plotted according to their individual GDP ranking, and their regional antibiotic consumption level. We collapsed countries into six ordered categories based on their level of increase or decrease in antibiotic use. The categories were: 50% + increase; 30–50% increase; 10–29% increase; neutral (less than 10% increase/decrease), 10–29% decrease, and 50% + decrease.

Inverse GDP ranking was plotted on a Cartesian graph such that the top-right corner of the graph theoretically contains countries with high influence and high self-interest in addressing AMR. Thresholds were chosen to delineate the four typology archetypes; for GDP, the most highly influential countries were designated as those within the wealthiest quartile globally; for change in antibiotic consumption countries were divided into two groups according to whether there had been an increase or decrease in antibiotic consumption over the 10-year span.

### Scoping review of barriers to action

A review of published and grey literature was conducted to identify and summarize barriers to early action on AMR. We initially identified key stakeholder groups, and tensions between these groups on AMR related issues (Additional file 1). Our identified barriers to early action on AMR were first classified according to the first four stages of the policy making cycle (agenda setting, agreement formulation, legitimation, and implementation) to show how early they would need to be addressed. Next, barriers were connected to any of the five relevant stakeholder groups: national governments, international organizations, civil-society organizations, the agricultural industry, and the pharmaceutical industry. In making these connections we identified relevant collaborators for action and gained insight into the choices a rational country might make given how AMR regulations would impact major industries and other stakeholders.

## Results

### Typology

**Fig. 1:**
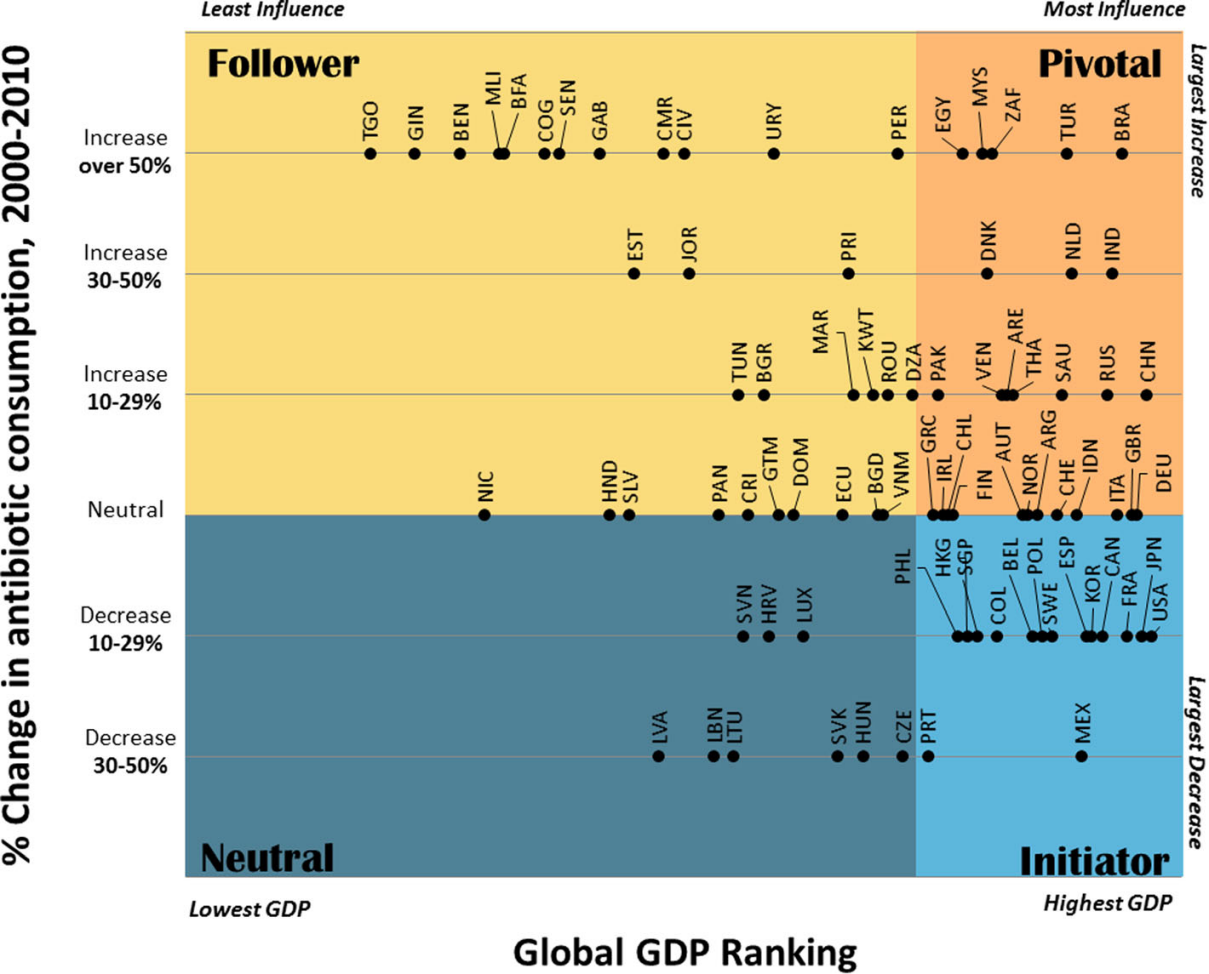
Global influence (GDP ranking) against self-interest in addressing AMR (10-year percent change in human antibiotic consumption). Four typology categories (Pivotal, Initiator, Follower and Neutral) representing different roles in addressing AMR are overlaid

We developed a four category typology of involvement in AMR based on data from 82 countries. In Fig. 1, we compared proxy measures for states’ global influence and self interest in addressing AMR across 82 countries and, based on the chart, developed four state archetypes which are overlaid on Fig. 1.

**Fig. 2:**
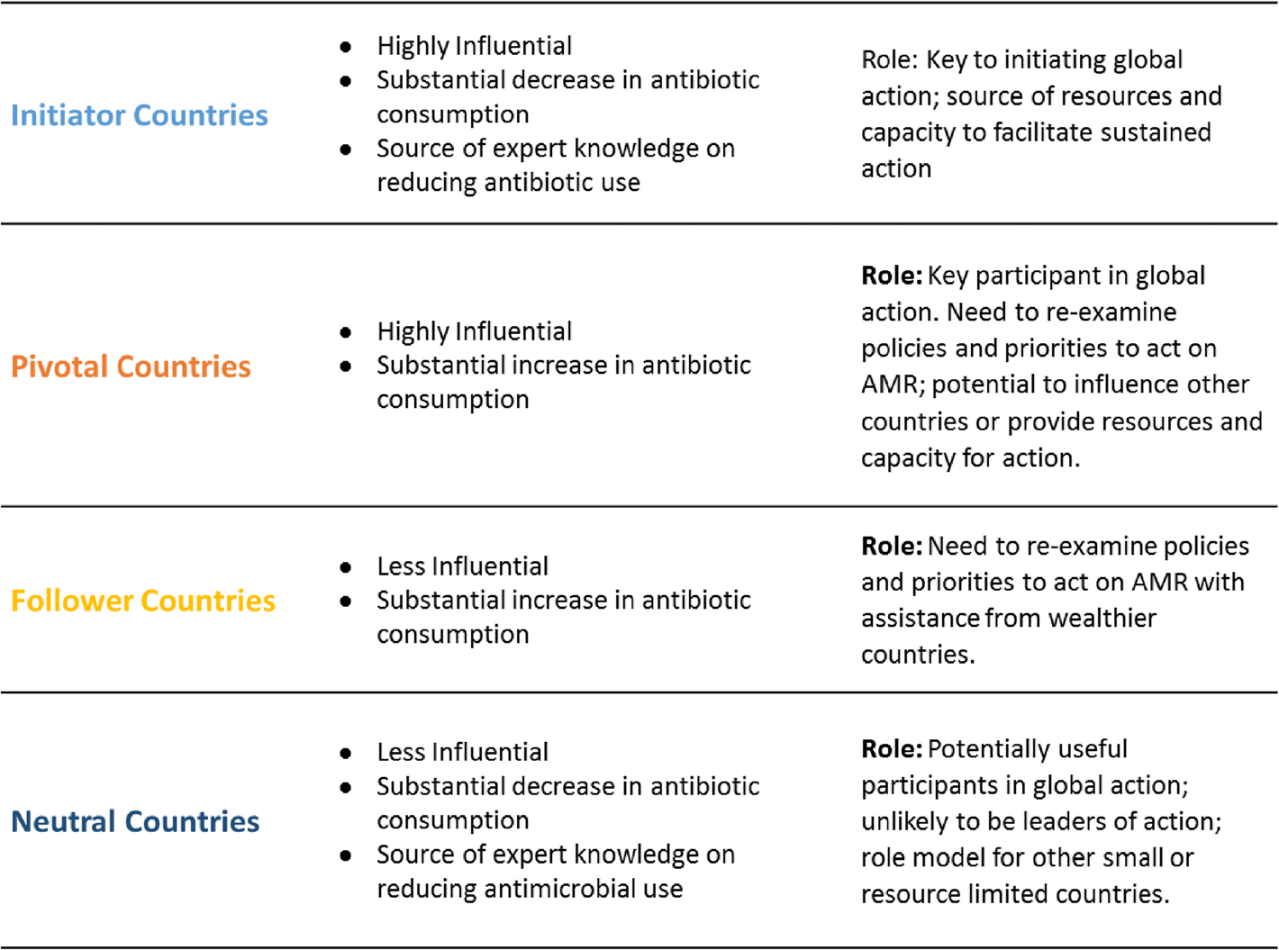
Description of typology categories

Based on this data we describe four simplified archetypes of countries (Fig. 2): 1) Initiator Countries, 2) Pivotal Countries, 3) Follower Countries, and 4) Neutral Countries.

Initiator Countries are both highly influential and saw substantial net decreases in human antibiotic consumption between 2000 and 2010. We theorize that these countries could be influential in initiating global action on AMR. Their success in decreasing antibiotic use makes these countries a potential source of expert knowledge on reducing antimicrobial use and AMR. Further, these countries are most likely to have the resources and capacity to facilitate sustained action on AMR.

Pivotal Countries are also highly influential with total GDPs falling within the top 25% globally. But these countries also saw some of the largest net increases in human antibiotic consumption between 2000 and 2010. Whether a particular Pivotal Country is likely to engage in coordinated global action on AMR depends on the internal context and dynamics within that country. However, the position of Pivotal Countries on the global influence scale suggests they could influence other countries’ positions and actions, and that Pivotal Countries ought to be involved in discussions on AMR. Further, the rising use of antibiotics in Pivotal Countries suggests a need to re-examine their policies on antimicrobial use. Examples of Pivotal Countries include all of the BRICS countries (i.e., Brazil, Russia, India, China, South Africa), Egypt, Saudi Arabia, and Thailand.

Follower Countries are less influential at the global level and saw net increases in human antibiotic consumption between 2000 and 2010. Countries in this group face a broad range of challenges, from basic surveillance and sanitation to the implementation of national action plans. Competing priorities mean that Follower Countries are not naturally disposed to support or commit resources to an AMR agreement. Nevertheless, countries who lack the resources to act independently on AMR may be incentivized to participate through collaboration with others and resource assistance. Potential resistance to action from Follower Countries is less of an immediate concern for establishing global AMR policies, because these countries are unlikely to dissuade other countries from participating.

Neutral Countries are less influential at the global level, and saw net decreases in human antibiotic consumption between 2000 and 2010. These countries might also be a source of expert knowledge, and might already be predisposed to participate in an AMR agreement. Countries in this group present the lowest risk if not initially included in an AMR agreement, because their antibiotic usage has decreased recently and few are major economic players.

### Barriers to AMR action

We identified several barriers to collective action on AMR that must be considered when crafting an international agreement (Table 1 or refer to Additional file 1 for more detail). A strong commitment to AMR is necessary from the outset, as competing health priorities may pose a barrier to keeping AMR front and central on the policy agenda (Table 1). Naturally, short-term emergencies will push long-term health threats to the background; political turmoil and other disease threats both compete with AMR for attention from policy makers [26]. There is also an ongoing lack of comprehensive data that creates uncertainty as to the scope of the AMR problem in most countries [5, 26, 27]. Establishing the scope of this problem will be key, yet many countries lack the infrastructure to carry out surveillance of either antimicrobial consumption or antimicrobial resistance. Further, international discord on surveillance practices makes producing reliable AMR data even more difficult [5]. Uncertainty as to what constitutes therapeutic or nontherapeutic antimicrobial use in livestock makes it difficult to assess the extent to which these practices exist in agriculture [28]. To measure the magnitude and scope of the problem, countries need adequate surveillance of AMR in humans, animals, and of antibiotic sales and prescribing practices [5].

**Table 1:** Barriers to Global Action on AMR by Policy Stage

Policy Stage	Barrier	Associated Stakeholders
Agenda Setting	Lack of Data	National Governments
		Supranational Organizations
		Civil Society Organizations
		Agricultural Industry
		Pharmaceutical Industry
	Achieving Public Engagement	National Governments
		Civil Society Organizations
	Competing Health Priorities	National Governments
		Pharmaceutical Industry
Agreement Formulation	Unknown Impact of AMR Control Policies	National Governments
		Supranational Organizations
		Civil Society Organizations
		Agricultural Industry
		Pharmaceutical Industry
	Reconciling Mandates, Business Models, and Perspectives of Key Stakeholders	National Governments
		Supranational Organizations
	Cost Distribution & Funding	National Governments
		Supranational Organizations
		Pharmaceutical Industry
Agreement Legitimation	Lack of Enforcement of International Law	National Governments
		Supranational Organizations
		Civil Society Organizations
		Agricultural Industry
		Pharmaceutical Industry
	Enacting Binding Rules on a Global Scale	National Governments
		Supranational Organizations
	Fragmentation of Global Health Governance and Initiatives	Supranational Organizations
Agreement Implementation	Unregulated Distribution of Antimicrobials (legal and counterfeit)	National Governments
		Civil Society Organizations
		Pharmaceutical Industry
	Lack of Capacity and Infrastructure	National Governments
		Civil Society Organizations
		Agricultural Industry
		Pharmaceutical Industry
	Reconciling Domestic Political Powers	National Governments
	Coordinating Actors	National Governments
		Supranational Organizations

A lack of data may also hinder AMR policy formulation (Table 1) [28]. To date, there have been insufficient efforts to evaluate the impact of existing AMR control policies, which creates a practical challenge to crafting and estimating the effect of new policies. Larger-scale evaluations or comparative effectiveness studies (e.g., for best farming practices) [29] would help determine the most effective provisions to include in an international agreement. Many of the evaluations that currently exist are single hospital interventions, which are typically analyzed only for economic impact at the hospital level [30].

The need to secure funding from external donors may also constrain policy formulation (Table 1). Where other nations or Civil Society Organizations (CSOs) place conditions on grants, policies may shift to meet those conditions. Reconciling key stakeholder mandates, business models, interests, and perceptions presents a further challenge. Policy makers may face strong opposition when highly organized industries expect to bear the highest cost of a policy [22]. For example, the agricultural industry will not likely support an international AMR agreement targeting agricultural use of antibiotics unless other cost-effective alternatives are found [29]. Likewise, pharmaceutical innovators are unlikely to support an international AMR agreement without incentives to invest in research and development (R&D).

Additionally, reconciling complex international regulatory standards in a way that encourages stakeholders to participate may be challenging. For example, the typically complicated and expensive regulatory requirements for developing new antimicrobials currently act as a deterrent to innovation [31].

Garnering legal support for an international AMR agreement presents further challenges (Table 1). A legally binding and enforceable agreement could ensure certain AMR policies are adopted and implemented on a global scale [16]. However, few entities have the authority and capacity to enact binding rules on a potentially global scale. International law can also be difficult to enforce. For example, countries can ratify legally binding agreements while opting-out of particular commitments or submitting reservations [32]. Addressing AMR at a global level will require mechanisms for achieving widespread implementation, compliance, and accountability [18].

Fragmentation of existing AMR efforts may also create barriers to policy legitimation. Some transnational entities have proposed norms and standards to respond to AMR and any new agreement will likely face challenges in harmonizing existing efforts and frameworks while addressing national and regional participation and needs [33].

To implement an international AMR agreement (Table 1), actors and stakeholders will need to continue to cooperate at national and international levels. As with policy formulation, the challenge will be to reconcile stakeholder interests as well as international regulatory standards in a manner that permits sustainable commitment to AMR policy. Similarly, countries may face challenges reconciling domestic powers to implement international standards, particularly in federal countries where powers over health are divided with domestic states or provinces [34, 35]. Successful implementation will also require enforcement and accountability mechanisms [18].

Weak infrastructure and capacity in research, surveillance, manufacturing, sanitation, and infection control may also hinder policy implementation. For example, research infrastructure is lacking for antimicrobial R&D due to poor financial incentives and past perceptions that research was no longer needed in this field [31]. Further, many countries lack the capacity to enforce laws against counterfeit pharmaceuticals [36]. At the most basic level, sanitation and infection control remain a problem in many places, and antibiotics are used as a stopgap to ensure patient safety [27].

## Discussion

### Principal findings

**Fig. 3:**
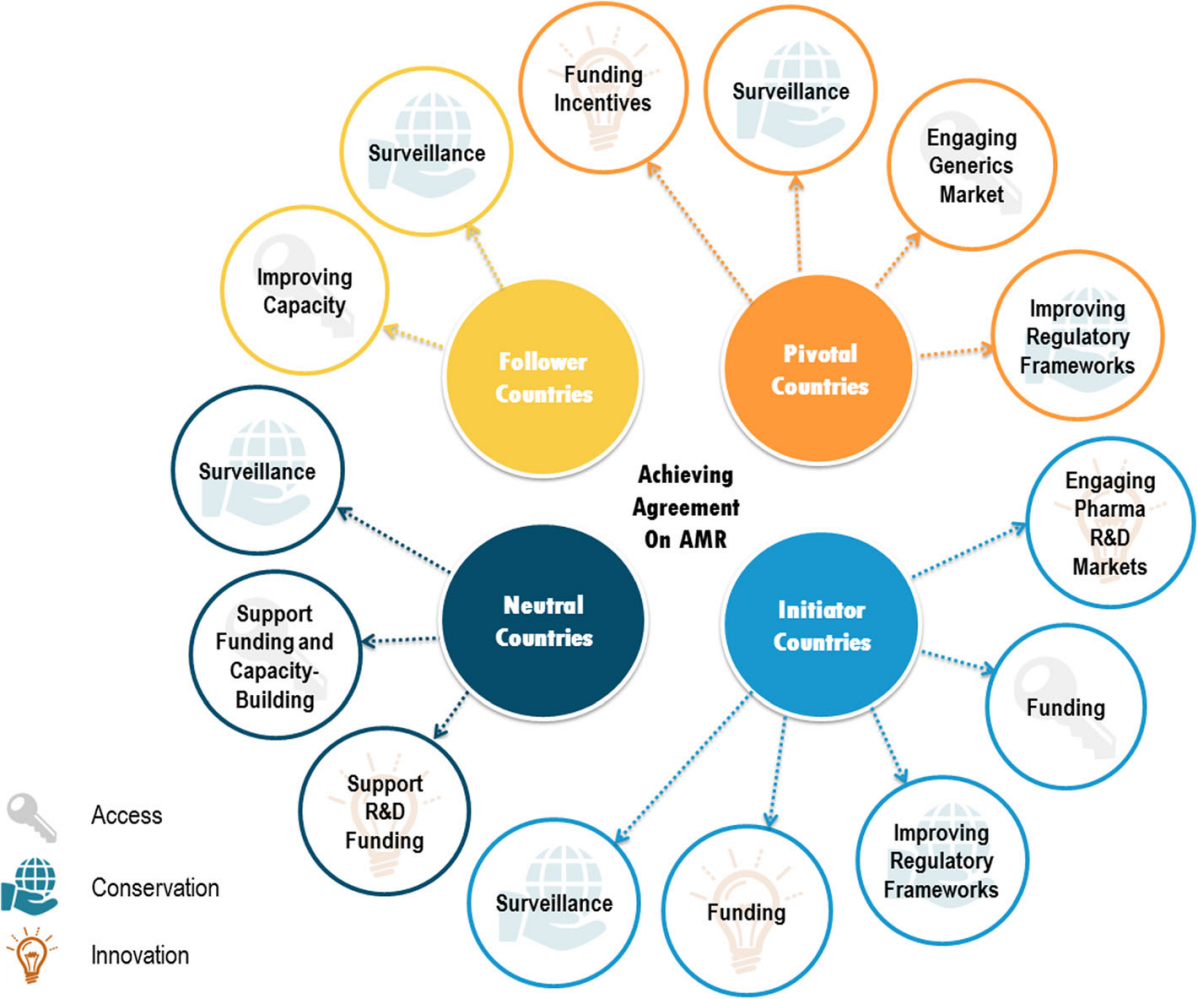
Roles of different types of countries in addressing AMR access, conservation and innovation. Pictograms from The Noun Project, created by Egor Culcea, Icon 54, and Korawan. M

We found that countries can be grouped into four archetypes, each with a different role to play in global action on AMR based on their global influence and self-interest in AMR. Figure 3 shows the areas where each type of country can be meaningfully engaged in action on AMR. Initiator and Pivotal Countries can take useful action in the most areas due to their wealth and capacity, their relationship with pharmaceutical industries, and their ability to provide foreign aid to countries with less capacity.

A large number of barriers must be resolved to achieve global collective action on AMR. For this reason, we project that a single broad global agreement that addresses all three areas of AMR action (i.e., access, conservation, and innovation) will not be easy to achieve. Many countries face a lack of resources and capacity, and a large number of competing domestic priorities. In these places, it would be difficult to even get AMR on the political agenda, much less the resources necessary to implement AMR policies. In wealthier countries there are numerous stakeholder perspectives that must be considered, and several other global actors to be engaged to support action. However, despite these challenges, we believe that there are mechanisms to engage the support of core-groups of countries in AMR action, which could be scaled up to achieve broad ranging global action.

### Policy implications

Based on our typology, we need not engage all countries in order to lay the groundwork for an agreement, particularly for more targeted agreements. Two groups, Initiator and Pivotal Countries, need to lead action on AMR. These countries have significant financial resources to contribute to capacity building in poorer countries, and many rank among the largest users of antimicrobials in both humans and animals [27].

The most politically feasible option in our view is to leverage smaller groups of specialized Initiator and Pivotal Countries to drive agreement, particularly in the early stages of agenda setting and policy development. Recognizing that eventually it will be necessary to attract participation from many nations and to widely implement policies, we believe that some agreements may be best served by resolving differences among a small group of countries with similar interests and priorities. For example, conservation in agriculture could be first addressed among countries with the largest agricultural export sectors. Having achieved a measure of consensus among this group, the agreement could then be modified to meet the needs of additional countries. Many Follower Countries will need to build substantial capacity before they are capable of participating in an agreement. These capacities include laboratory capacity for implementing surveillance and testing measures including human resources, and improvements to regulatory measures, and enforcement. We encourage the participation of Follower Countries in discussions to build international agreement, and encourage their eventual integration into a proposed agreement. We suggest that Follower Countries be engaged as observers of the process, giving them the ability to participate in policy discussions while building the capacity for implementation. Follower countries would gain flexibility through this process, as they would not be subject to immediate financial or resource commitments, and could work alongside other nations towards a gradual integration into an agreement.

Several strategies are available to increase participation in an agreement. Broader coordinated action can be increased using incentives and trade-offs to gain agreement from countries outside the core group of leading countries. For example, Pivotal Countries with an emerging pharmaceutical market could be engaged to support access to affordable antimicrobials in resource-poor regions in exchange for contributing to a global pool for funding innovation. This type of measure would alleviate the financial burden placed on Initiator Countries who are driving innovation, and thereby facilitate access in lower-income countries—even if they are not part of an initial AMR agreement. Alternatively, lower-income Pivotal Countries could be offered access to research funding and new innovations in exchange for adopting and implementing conservation policies, which could facilitate agreement on innovation and conservation [16]. Relying on certain Initiator and Pivotal Countries to initiate a global solution to AMR places some pressure on those countries from a resource perspective. However, the potential benefits of taking the lead outweigh the costs. Because AMR is a global issue, each country stands to benefit when other countries take action. If high-influence countries can initiate global AMR policies, Follower and Neutral Countries will be easier to attract. Each country has traditionally been responsible for its own domestic policies on a variety of health issues, but given the cross-border, global nature of AMR, the onus has now become a collective one. To enact sustainable solutions on a global scale, countries with the highest level of influence must take the lead and advocate for united global action.

There is also a role for non-governmental stakeholders in this phased-in approach to an international agreement. Supranational organizations, for example, can offer a platform for discussions where countries can come together to develop an agreement along issue specific regional lines. Civil society organizations can provide expertise and support, particularly for the recruitment of Follower Countries into an agreement. GARP, the Global Antibiotic Resistance Partnership, is already acting in this capacity, bringing financing from civil society organizations together with national governments to improve capacity for responding to antimicrobial resistance [37].

### Strengths and weaknesses

Our typology provides an organizing structure that assists in discussing the similarities of groups of countries. We have taken a comprehensive approach to deriving our classification system; the typology is transparent and has been developed based on data, rather than preconceived stereotypes of countries. In using data from 82 countries, we have sacrificed some nuance, particularly in describing countries that fall on the borderlines of our categories. We recognize that there are a number of reasons why some countries sit on the neutral line with no major variation in antibiotic consumption over 10 years; some may have acted to curb their antibiotic consumption before 2000 and others may have taken no action at all. These countries should be analyzed on a case-by-case basis to determine which type classification is most appropriate. We also recognize that, in the long term, countries will not remain in the same category that they currently occupy in Table 1. Our antibiotic consumption data was from 2000 to 2010; as data becomes available for more recent years, countries that substantially increase or decrease their antibiotic consumption should be re-evaluated accordingly.

We were unable to obtain data for this project on agricultural use of antibiotics due to a global lack of data on this topic. Agricultural use of antimicrobial agents is a contentious issue for many countries with strong agricultural industries and policies to limit use in animals would undoubtedly influence the way such countries participate in AMR agreement. We recognize this is a shortcoming of our categorization system. Globally it is necessary to improve reporting on antibiotic consumption in agriculture, and to develop a measurement system that allows for comparison across animal species.

### Future research directions

Currently, there are few proposals that suggest concrete steps for global action on AMR. As new proposals are developed, they should take into account the archetypes described here to determine whether they have support from relevant Initiator and Pivotal Countries. Additionally, further research is needed to find strategies for addressing many of the barriers we have identified. Improvements in capacity, surveillance, and regulation will go a long way towards making global collective action more feasible.

## Conclusions

Achieving a global agreement on access, conservation, and innovation will not be easy – as we have shown there are several barriers that must be overcome. However, smaller core groups of interested Initiator and Pivotal Countries could develop AMR policies and resolve many of these barriers. If highly influential countries take the lead, agreements could then be scaled up to achieve global action.

## Additional file

Additional file 1: Detailed description of methods and results is available in the Full Report/Web 117 Appendix. (PDF 8194 kb)

## Abbreviations

AMR: Antimicrobial Resistance
CDDEP: Center for Disease Dynamics, Economics & Policy
CSOs: Civil Society Organizations
GDP: Gross Domestic Product
LMIC: Low and Middle Income Countries
R&D: Research and Development

## References

[1] International Federation of Pharmaceutical Manufacturers & Associations (2015). Rethinking the Way We Fight Bacteria.

[2] Public Health Agency of Canada. The Chief Public Health Officer’s Report on the State of Public Health in Canada, 2013: Infectious Disease - The Never-ending Threat. Ottawa: Public Health Agency of Canada; 2013.

[3] Barlam TF, Gupta K (2015). Antibiotic resistance spreads internationally across borders. J Law Med Ethics.

[4] Review on Antimicrobial Resistance. Antimicrobial Resistance: Tackling a crisis for the health and wealth of nations United Kingdom 2014 [cited 2015 Nov 15]. Available from: http://amr-review.org/home.

[5] World Health Organization. Antimicrobial Resistance - Global Report on Surveillance. In: Organization WH, editor. Geneva: WHO Press; 2014. http://www.who.int/drugresistance/documents/surveillancereport/en/

[6] Hoffman SJ, Outterson K (2015). What will It take to address the global threat of antibiotic resistance?. J Law Med Ethics.

[7] Hollis A, Maybarduk P (2015). Antibiotic resistance is a tragedy of the commons that necessitates global cooperation. J Law Med Ethics.

[8] Daulaire N, Bang A, Tomson G, Kalyango JN, Cars O (2015). Universal access to effective antibiotics is essential for tackling antibiotic resistance. J Law Med Ethics.

[9] World Health Organization (2014). Resolution WHA 67.25: Antimicrobial resistance.

[10] World Health Organization (2001). WHO Global Strategy for Containment of Antimicrobial Resistance.

[11] Organization WH (2007). Resolution WHA60.16: Progress in the rational use of medicines.

[12] World Health Organization (2001). Resolution WHA54.11: WHO medicines strategy.

[13] Organization WH (2007). Progress Report: WHA A60/28 - Improving the containment of antimicrobial resistance, 5 April 2007.

[14] World Health Organization (2015). Global Action Plan on Antimicrobial Resistance.

[15] The Lancet Infectious Diseases (2016). Time for global political action on antimicrobial resistance. Lancet Infect Dis.

[16] Behdinan A, Hoffman SJ, Pearcey M (2015). Some global policies for antibiotic resistance depend on legally binding and enforceable commitments. J Law Med Ethics.

[17] Hoffman SJ, Caleo GM, Daulaire N, Elbe S, Matsoso P, Mossialos E (2015). Strategies for achieving global collective action on antimicrobial resistance. Bull World Health Organ.

[18] Hoffman SJ, Ottersen T (2015). Addressing antibiotic resistance requires robust international accountability mechanisms. J Law Med Ethics.

[19] Ardal C, Outterson K, Hoffman SJ, Ghafur A, Sharland M, Ranganathan N (2016). International cooperation to improve access to and sustain effectiveness of antimicrobials. Lancet.

[20] World Health Organization. United Nations high-level meeting on antimicrobial resistance 2016 [cited 2016 Oct 16]. Available from: http://www.who.int/antimicrobial-resistance/events/UNGA-meeting-amr-sept2016/en/.

[21] Chioro A, Coll-Seck AM, Hoie B, Moeloek N, Motsoaledi A, Rajatanavin R (2015). Antimicrobial resistance: a priority for global health action. Bull World Health Organ.

[22] Reich M (2002). The politics of reforming health policies. IUHPE - Prom & Ed.

[23] Putnam R (1988). Diplomacy and domestic politics: the logic of Two-level games. Int Organ.

[24] World Bank (2014). GDP Ranking: World Bank.

[25] The Centre for Disease Dynamics EP. Methodology: Antibiotic Use [cited 2016 Feb 20]. Available from: https://resistancemap.cddep.org/MethodologyAU.php.

[26] World Health Organization. Worldwide Country Situation Analysis: Response to Antimicrobial Resistance. Geneva: World Health Organization; 2015.

[27] Centre for Disease Dynamics Economics & Policy (2015). State of the World’s Antibiotics, 2015.

[28] Landers TF, Cohen B, Wittum TE, Larson EL (2012). A review of antibiotic use in food animals: perspective, policy, and potential. Public Health Rep.

[29] Grace D. Review of Evidence on Antimicrobial Resistance and Animal Agriculture in Developing Countries. UK: International Livestock Research Institute; 2015.

[30] Smith RD, Yago M, Millar M, Coast J (2006). A macroeconomic approach to evaluating policies to contain antimicrobial resistance: a case study of methicillin-resistant Staphylococcus aureus (MRSA). Appl Health Econ Health Policy.

[31] International Federation of Pharmaceutical Manufacturers & Associations. IFPMA Position on Antimicrobial Resistance (AMR) 2011 [cited 2015 Nov 15]. Available from: http://www.ifpma.org/wp-content/uploads/2016/02/IFPMA_Position_on_Antimicrobial_Resistance_7April2011.pdf.

[32] Kates J, Katz R (2011). The role of treaties, agreements, conventions, and other international instruments in global health. Infect Dis Clin North Am.

[33] Abdula N, Macharia J, Motsoaledi A, Swaminathan S, VijayRaghavan K (2016). National action for global gains in antimicrobial resistance. Lancet.

[34] Wilson K, McDougall C, Upshur R, Joint Centre for Bioethics SGHERG (2006). The new International Health Regulations and the federalism dilemma. PLoS Med.

[35] Fidler DP (1998). Legal issues associated with antimicrobial drug resistance. Emerg Infect Dis.

[36] Ratanawijitrasin S, Wondemagegnehu E. Effective Drug Regulation: A Multicountry Study. Geneva: World Health Organization; 2002.

[37] Center for Disease Dynamics Economics & Policy. Global Antibiotic Resistance Partnership 2015 [cited 2015 Oct]. Available from: http://www.cddep.org/garp/home#sthash.vcxp8c1t.dpuf.

