# Supplementary material for: Developing an approach to assessing the political feasibility of global collective action and an international agreement on antimicrobial resistance
Source: Glob Health Res Policy. 2016 Dec 13;1:20. doi: 10.1186/s41256-016-0020-9 (PMC5693542; doi:10.1186/s41256-016-0020-9)
Supplement: Additional file 1: — Detailed description of methods and results is available in the Full Report/Web 117 Appendix. (PDF 8194 kb) [file 41256_2016_20_MOESM1_ESM.pdf]

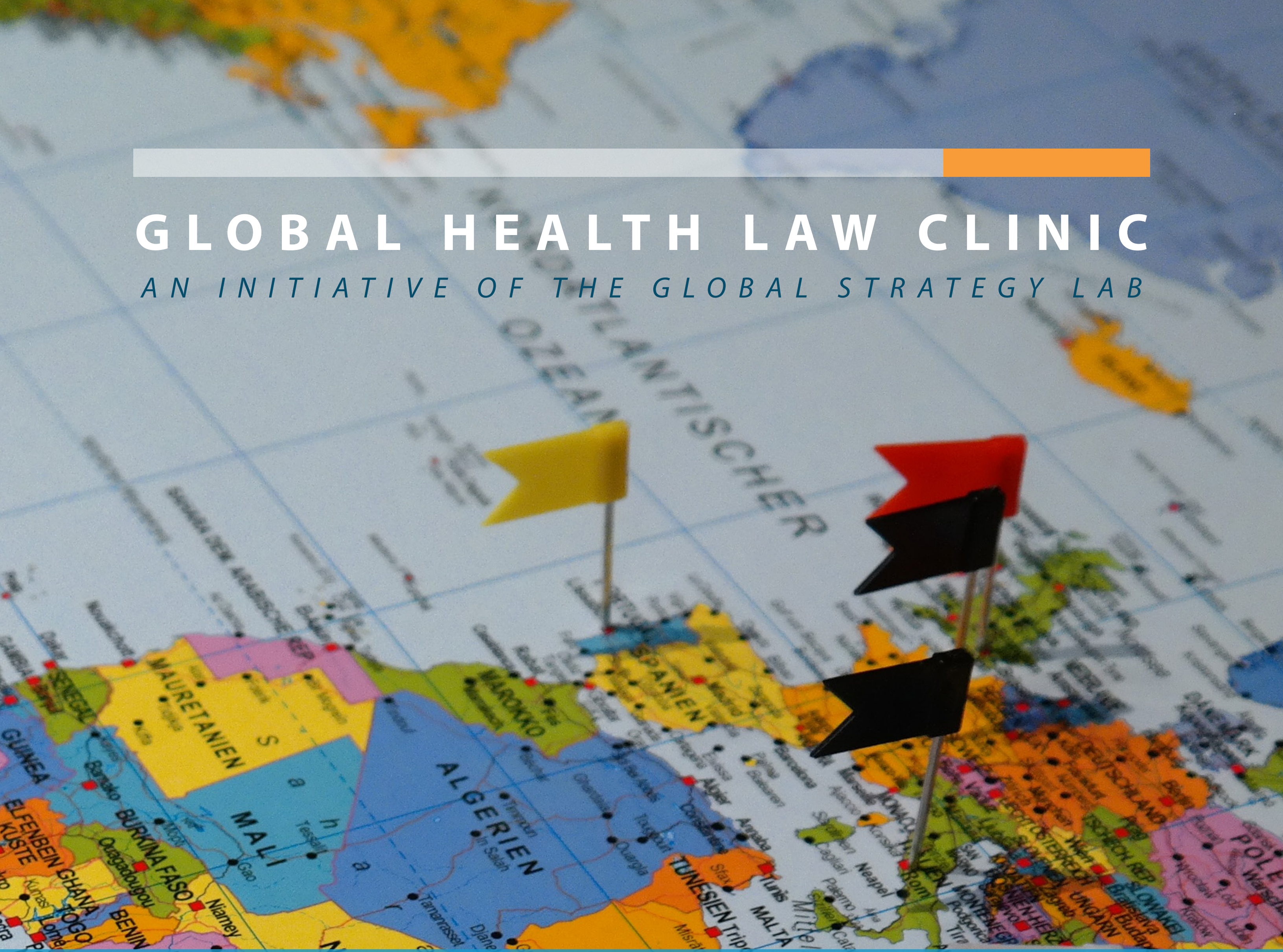

GLOBAL HEALTH LAW CLINIC

AN INITIATIVE OF THE GLOBAL STRATEGY LAB

Assessing the Political Feasibility of an International Agreement on Antimicrobial Resistance

Marie Évelyne Danik, Ioana Pantis, Susan Rogers Van Katwyk and Rachel Smith

Edited by Steven J. Hoffman and Lathika Sritharan

uOttawa

Labo de stratégie mondiale
au Centre de droit, politique et éthique de la santé de uOttawa

Global Strategy Lab
at the uOttawa Centre for Health Law, Policy and Ethics

Global Health Law Clinic Publication Series
Assessing the Political Feasibility of an
International Agreement on Antimicrobial Resistance

April 2016

ISBN 978-0-9951601-1-8 (online)
ISSN 2371-137X (online)

Suggested citation for the report

Danik ME, Pantis I, Rogers Van Katwyk S, Smith R. Assessing the Political Feasibility of an International Agreement on Antimicrobial Resistance. In: Hoffman SJ, Sritharan L. (eds.) *Global Health Law Clinic Publication Series*. Ottawa, Canada: Global Strategy Lab, University of Ottawa; 2016. Available from: www.globalstrategylab.org/clinic/reports/feasibility-of-amr-agreement-2016.pdf.

© Global Strategy Lab 2016

Contents of this publication may be reproduced in whole or in part provided the intended use is for non-commercial purposes and full acknowledgement is given to the authors and editors and to the Global Strategy Lab as publisher. Reasonable precautions have been taken to verify the information contained in this publication. However, the published material is being distributed without warranty of any kind, either expressed or implied. The publication should particularly not be relied upon for legal advice. The responsibility for interpretation and use of the material lies with the reader. In no event shall the authors, editors or publisher be liable for damages arising from its use.

This publication was commissioned by the Norwegian Institute of Public Health. Funding for this publication was provided by the Global Strategy Lab. The views expressed here do not necessarily represent the views of the Norwegian Institute of Public Health, Global Strategy Lab or its partners.

Graphic design and post-production by Tom Jacobs | www.moodmarketing.ca
Cover image: CC0 public domain photo by Hans.

About the Global Health Law Clinic | www.globalstrategylab.org/clinic
The Global Health Law Clinic is an experiential learning opportunity for students to apply their previous studies to real-world global health practice. Students work in teams to provide a United Nations agency, government, or civil society partner with research, analysis and advice on addressing a pressing global health challenge facing them.

About the Global Strategy Lab | www.globalstrategylab.org
The Global Strategy Lab, directed by Steven J. Hoffman, is an interdisciplinary research program based at the University of Ottawa's Faculty of Law. The Lab brings cutting-edge science and scholarship to bear on how global institutions, instruments and initiatives are designed to better address transnational health threats and social inequalities.

TABLE OF CONTENTS

Executive Summary 4

Introduction 6

State Actor Typology 7

Influential Stakeholders 10

Barriers to International Agreement 14

Political Feasibility Analysis 19

Conclusion 28

References 29

Appendix 36

EXECUTIVE SUMMARY

Antimicrobial resistance (AMR) is a growing international health threat that calls for global collective action.[1] An international agreement committing states to act on AMR could spur the necessary global response using a variety of mechanisms, instruments, and forums.[2,3] Whether states would likely support and implement such an AMR agreement depends on the extent to which proposed policies serve domestic, international, and stakeholder interests.[4,5] States and stakeholders must perceive that the benefits of the agreement outweigh the costs for an agreement to be politically feasible.

This report explores the political feasibility of an international AMR agreement. We identify key stakeholder interests that may impact global cooperation on AMR and assess barriers to agreement at the early stages of the policymaking process. Using a country typology based on international influence and interest in AMR, we analyze options for achieving agreement on antimicrobial access, conservation, and innovation. Our goal is to provide policymakers with a framework for navigating the political feasibility of global agreement on AMR.

To assess political feasibility, we categorize countries into four types based on global influence and interest in AMR: Initiator Countries, Pivotal Countries, Follower Countries, and Neutral Countries. We assume that countries act rationally according to their best interests, taking into account their financial and resource capabilities, competing priorities, the perceived domestic and international threat of AMR, and key stakeholder interests.

While an agreement committing all countries to act simultaneously on access, conservation and innovation would ensure a comprehensive response to AMR, we conclude that such an agreement is not immediately politically feasible without additional incentives and supports. Resource and capacity limitations, competing priorities, and conflicting stakeholder perspectives prevent many from taking immediate action, and some barriers will take longer to overcome than others. Taking global action on surveillance is one way the world could begin to overcome these resource gaps while building a stronger foundation for agreements on other AMR responses.

To achieve long-term coordinated action on AMR, we recommend that core groups of specialized Initiator and Pivotal countries be engaged to create the basis of an agreement before inviting other countries to join. Early engagement by a smaller group of countries with the capacity to take action on access, conservation, and innovation will facilitate agreement, and provide a platform for creating policies capable of gaining global traction.

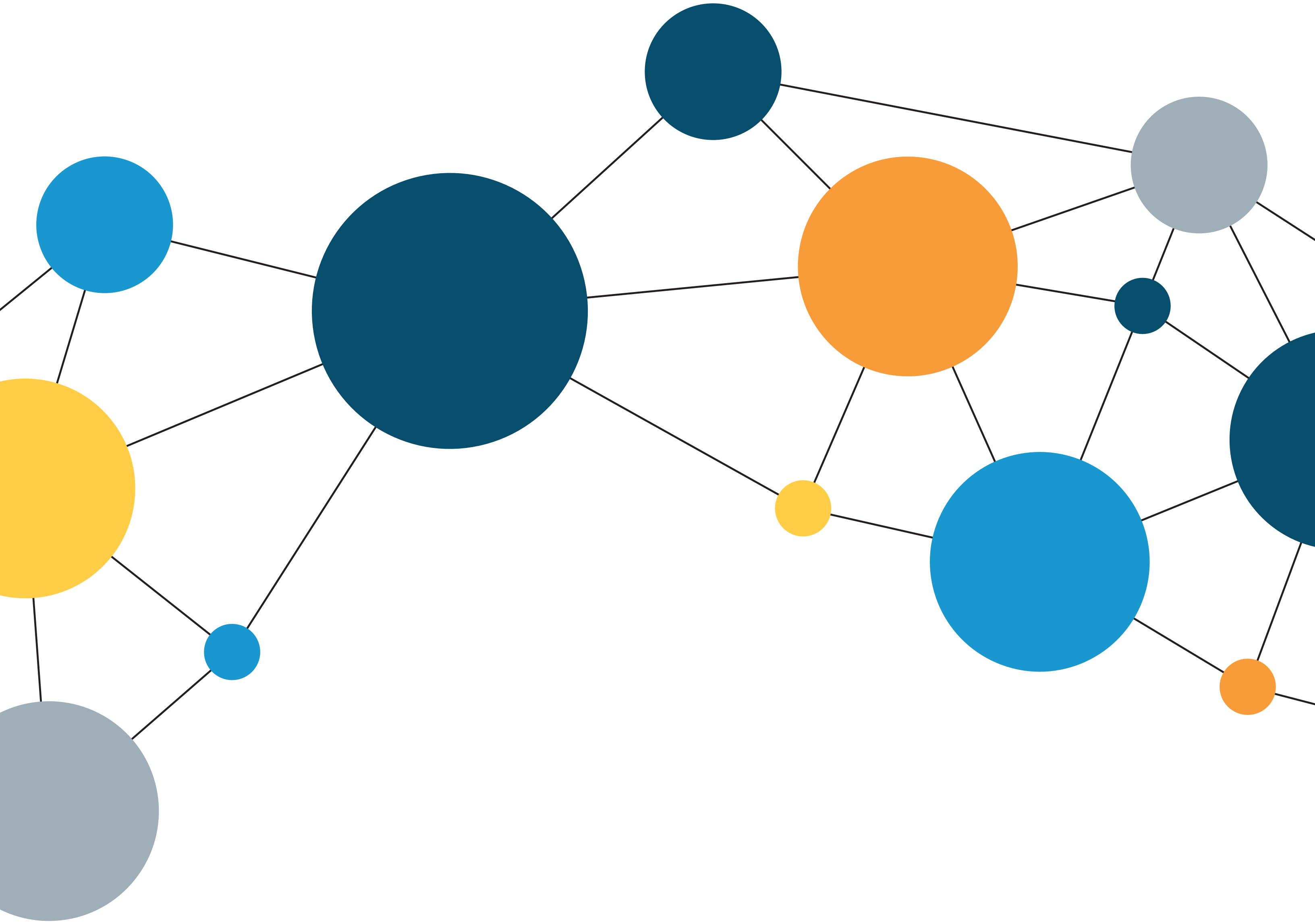

INTRODUCTION

AMR occurs when microorganisms such as bacteria, viruses, fungi, and parasites evolve to become resistant to antimicrobial drugs.[6,7] International travel, agricultural practices, trade, and environmental contamination make it easy for resistant microbes to cross national borders.[8] Today, approximately 500,000 people worldwide die each year from AMR infections. By 2050, AMR could cause 10 million annual deaths and $100 trillion cumulative gross domestic product (GDP) loss.[9] Thus, AMR is a global issue that requires swift and coordinated action to prevent a post-antibiotic era.[7,8]

A comprehensive solution to AMR must address three interrelated concerns: (1) access; (2) conservation; and (3) innovation.[2] Inappropriate and excessive use of antimicrobials accelerates the development of AMR.[10] A lack of innovation means that stores of effective antimicrobials are depleting.[7] Meanwhile, millions of people die each year because they cannot access effective drugs for antimicrobial-susceptible infections.[11]

Hoffman and Outterson highlight the interdependencies between access, conservation, and innovation in a global AMR strategy.[2] Increased access without conservation and innovation depletes limited antimicrobial resources. Antimicrobial conservation inherently limits access to antimicrobials and reduces incentives for innovation.[2] However, antimicrobial innovation without conservation risks wasting the time and money invested in antimicrobial research and development (R&D), because irresponsible access accelerates resistance. Further, innovation is costly, but increasing the price of antimicrobials hinders access to novel medications for those in need.[2] These interdependencies are illustrated in Figure 1.

This study assesses the political feasibility of an international AMR agreement. We define political feasibility in this context as the likelihood that political and stakeholder interests can align to create and implement policies addressing antimicrobial access, conservation, and innovation. While an international agreement could initiate the necessary global action on AMR,[2,3] states are unlikely to support or implement such an agreement unless its provisions benefit domestic and stakeholder interests as well as international priorities.[4,5] To our knowledge, no such assessment has yet been done, but is a necessary first step to initiating global agreement to act on AMR.

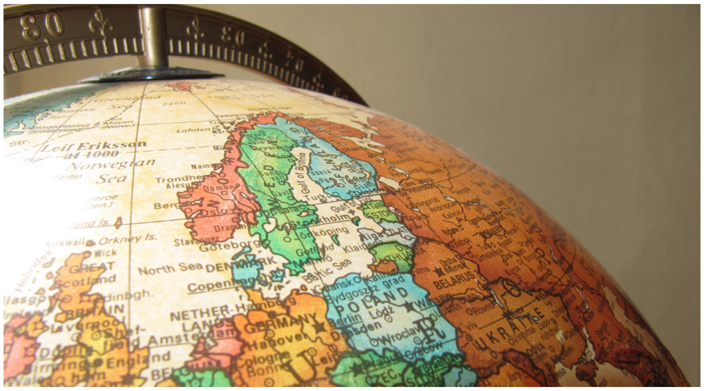

CC0 public domain photo by pixelcreatures

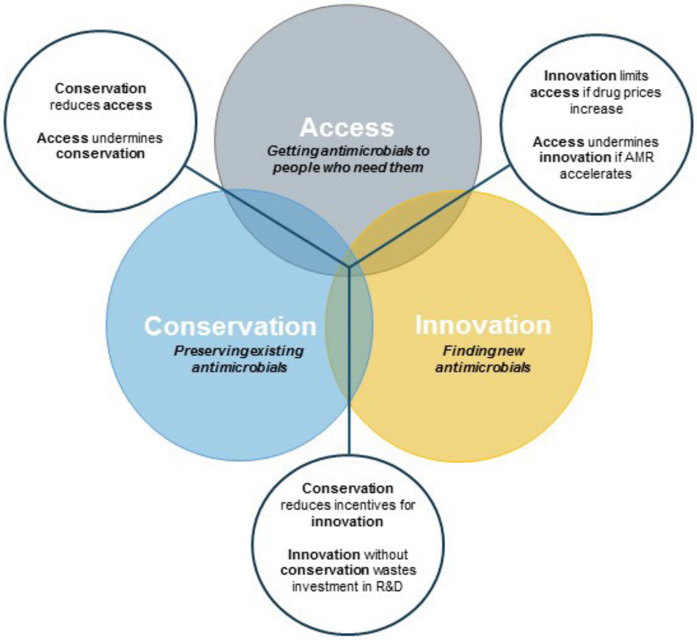

Figure 1 - Access, Conservation, and Innovation are Interrelated
Adapted from Hoffman and Outterson (2015)[2]

STATE ACTOR TYPOLOGY

To analyze political feasibility, we must consider how countries will act in the face of different proposed international agreements. Predicting how each country will act is a complex process because each country has its own considerations, history with AMR, and global standing. To facilitate our discussion, we describe four simplified types of countries that might be engaged in an AMR agreement: Initiator Countries, Pivotal Countries, Follower Countries and Neutral Countries. Categories were created on the basis of surrogate measures for two important factors: (1) global influence, and (2) interest in addressing AMR.

Global influence often depends on financial power. Thus, we used the World Bank's 2014 GDP rankings to categorize countries by global influence and designated the top 25% of countries by GDP as the most influential.[12]

To categorize countries based on interest in addressing AMR, we looked to antibiotic consumption. We assume that countries that have seen recent large increases in antibiotic consumption have a greater stake in combating AMR than countries with low or stable consumption rates. Yet, annual consumption rates, even standardized per capita, obscure underlying trends. For example, low rates of consumption

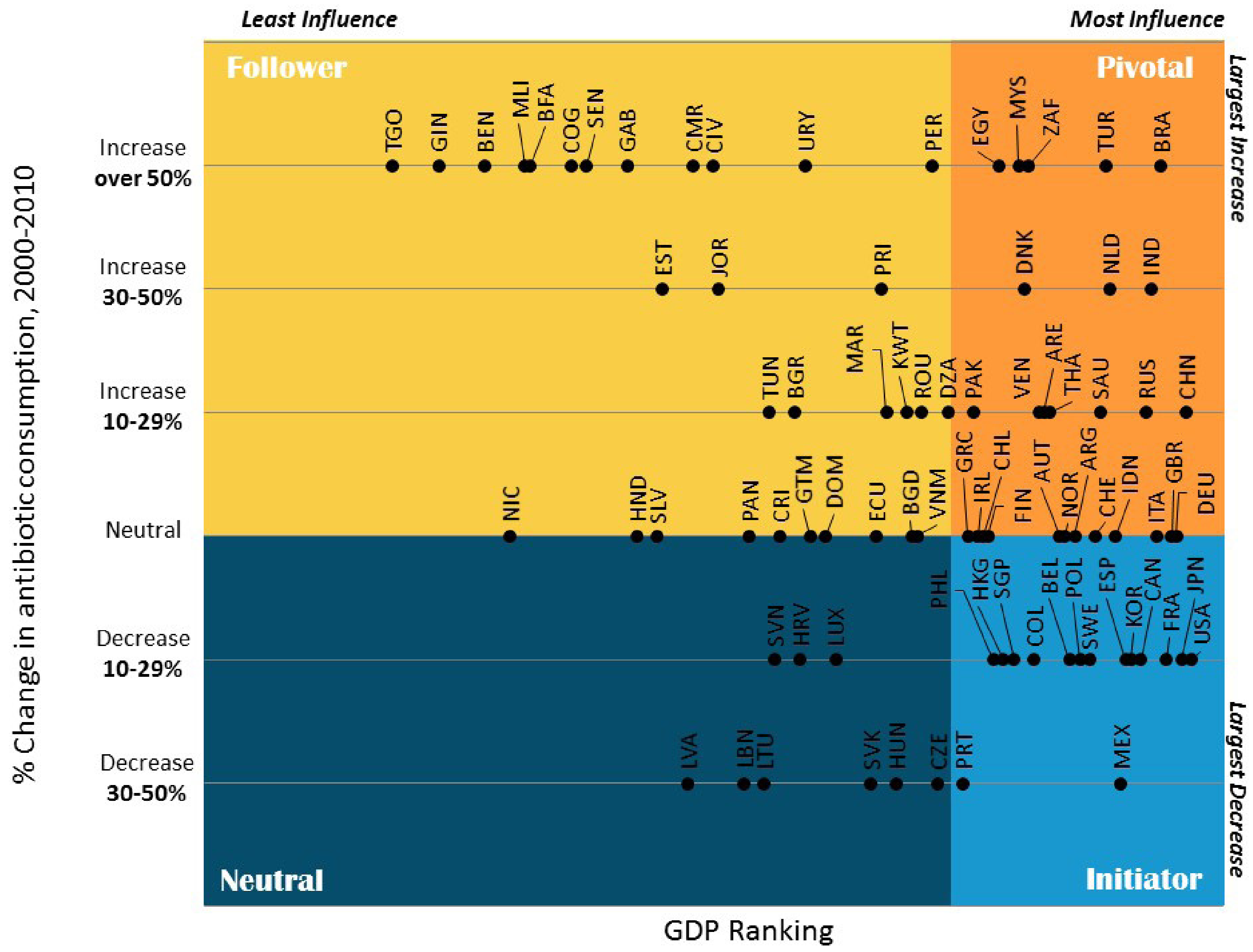

Figure 2 - Country Typology

might indicate either successful stewardship efforts or severe problems with antibiotic access. Evaluating annual rates might also be misleading due to short-term health and market shocks. To overcome these difficulties, we chose to use 10-year percent change in human antibiotic consumption as our indicator of country interest in addressing AMR, based on data obtained from the Center for Disease Dynamics, Economics & Policy (CDDEP). The CDDEP data was based on data collected in the IMS Health MIDAS database from 2000 to 2010.[13] This measure gives a long-term overview of AMR change at the national level. Whether the country had high or low consumption rates in 2000, a large proportional increase in consumption signals a need to re-examine national priorities, while a decrease indicates successful stewardship efforts.

We plotted GDP ranking against 10-year percent change in human antibiotic consumption for 82 countries and developed the four country types illustrated in Figure 2. Each country type is explained below, using example countries to illustrate key characteristics of countries in each grouping. Countries that rest on the horizontal axis had little or no change in human antimicrobial consumption between 2000 and 2010 for various reasons: some may have acted before 2000 and others may have taken no action at all. These countries should be analyzed on a case-by-case basis to determine which type classification is most appropriate. Our complete methods for creating our typology are provided in the Appendix.

1. Initiator Countries

Initiator Countries are both highly influential—their GDPs fall within the top 25% globally—and saw substantial net decreases in human antibiotic consumption between 2000 and 2010. We theorize that these countries could be influential in initiating global action on AMR. Their success in decreasing antibiotic use makes these countries a potential source of expert knowledge on reducing antimicrobial use and AMR. Further, these countries are most likely to have the resources and capacity to facilitate sustained action on AMR.
Mexico (MEX) is an example of an Initiator Country that has taken steps towards reducing the threat of AMR with some success. Over-the-counter (OTC) antibiotic sales were common practice in Mexico until 2010 when the country implemented policies to enforce existing laws on prescription requirements.[14] Research suggests that this policy change supported a decrease in total antimicrobial consumption in Mexico between 2007 and 2012.[14]

The United States of America (USA) and South Korea (KOR) are further examples of Initiator Countries that have prioritized and taken significant steps to combat AMR. Both countries have undertaken initiatives domestically, including national action plans and funding commitments, and have demonstrated a willingness to reach out to and collaborate with stakeholders and other nations.[15-19] In the USA, for example, the Food and Drug Administration successfully phased out non-treatment uses of medically important antibiotics in agriculture and plans to change OTC status, integrating industry perspectives in the process.[20-22] The agricultural industry in South Korea has also taken action.[23]

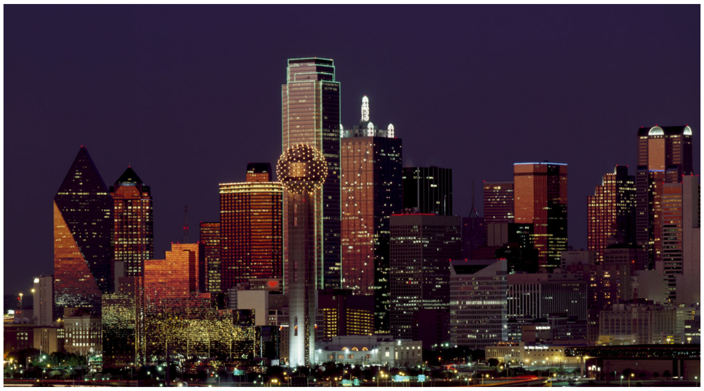

CC0 public domain photo by skeeze

2. Pivotal Countries

Pivotal Countries are also highly influential with total GDPs falling within the top 25% globally. But these countries also saw some of the largest net increases in human antibiotic consumption between 2000 and 2010. Whether a particular Pivotal Country is likely to engage in coordinated global action on AMR depends on the internal context and dynamics within that country. However, the position of Pivotal Countries on the global influence scale suggests they could influence other

countries' positions and actions, and that Pivotal Countries ought to be involved in discussions on AMR. Further, the rising use of antibiotics in Pivotal Countries suggests a need to (re)examine their policies on antimicrobial use. Examples of Pivotal Countries include all of the BRICS countries (i.e., Brazil, Russia, India, China, South Africa), Egypt, Saudi Arabia, and Thailand.

Pivotal Countries are often characterized by competing domestic priorities. Brazil (BRA) is an interesting example of a country that has taken some action on AMR, but that nevertheless may resist or face barriers to implementing AMR policies due to competing domestic pressures. Following an increase of more than 50% in human antibiotic consumption, Brazil implemented successful OTC regulations in response to deaths from multidrug resistant (MDR) hospital infections.[14] However, Brazil is also a major meat exporter that relies heavily on antibiotics to increase livestock production.[24] In this light, Brazil would face challenges in regulating livestock antibiotic use. Brazil may also be worried about any global action that could lead to restrictions on access to non-patented generic antibiotics or negatively affect the generic medicines industry.

Egypt (EGY) and Saudi Arabia (SAU) are further examples of Pivotal Countries with competing priorities that could impact their interest in engaging in global action on AMR.
Egypt has successfully improved surveillance of hospital AMR infections through a partnership with the WHO and with expertise from the USA; however, AMR rates remain high.[25] That pharmaceutical laws are not strictly enforced contributes to the AMR problem in both Egypt and Saudi Arabia, where pharmacists also a have financial interest in antibiotic sales.[26-28] Saudi Arabia's AMR problem is compounded by the fact that the country lacks a national action plan and awareness or understanding of the AMR issue,[21] and the annual Hajj pilgrimage risks accelerating the spread of AMR.[29] Nevertheless, an international AMR agreement would benefit from having Saudi Arabia's support, given the country's influence in the Middle East.

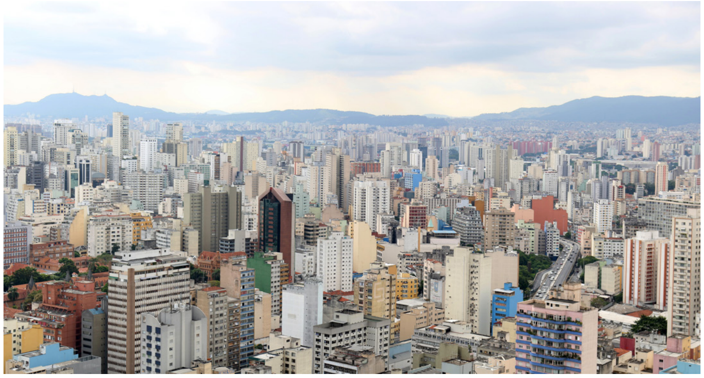

CC0 public domain photo by joelphotos

3. Follower Countries

Follower Countries are less influential at the global level and saw net increases in human antibiotic consumption between 2000 and 2010. Countries in this group face a broad range of challenges, from basic surveillance and sanitation to the implementation of national action plans. Competing priorities mean that Follower Countries are not naturally disposed to be supporters of an AMR agreement. Potential resistance from Follower Countries is less of an immediate concern for establishing global AMR policies, because these countries are unlikely to dissuade other countries from participating. Nevertheless, as many Follower Countries may not have the capacity or resources to act on AMR, financial assistance may incentivize these countries to engage in the global response.

Romania (ROU) and Vietnam (VNM) are Follower Countries that have made progress on AMR with assistance from foreign donors, though they still face challenges. Romania developed a national action plan to improve stewardship, surveillance of hospital infections, and prevention measures with grants from Norway and the EEA.[30] While some steps at regulation are taking place, non-prescription antibiotic use in Romania remains high.[31] In 2013, Vietnam became the first country in the Western Pacific Region to launch a national action plan to tackle AMR and improve national stewardship capacity.[32,33] Though Vietnam falls on the horizontal axis in our typology, we classify it as a Follower Country because AMR efforts in this country have been bolstered by partnerships with external parties.[31,34] Still, Vietnam continues to face challenges with

antimicrobial use in animals,[35] as regulations focus primarily on products for export rather than the domestic market.[32]

Countries in French West Africa (BEN, BFA, CIV, CMR, COG, GAB, GIN, MLI, SEN, TGO) face many barriers to acting on AMR without support. A lack of knowledge about local conditions is the biggest barrier. There is awareness that some AMR infections—such as MDR-TB—are a concern for the region, yet weak laboratory infrastructure means these countries lack drug resistance surveillance data.[36] Further, the lack of regulations and poor enforcement means that antibiotics—which are often of poor quality—can be dispensed from pharmacies and street vendors.[37] A great deal of capacity building would be required for these countries to take part in global AMR efforts.

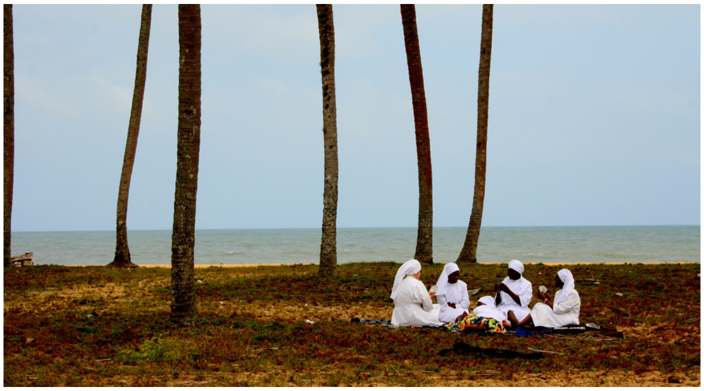

CC0 public domain photo by Anne-Onyme

4. Neutral Countries

Neutral Countries are less influential at the global level, and saw net decreases in human antibiotic consumption between 2000 and 2010. These countries might also be a source of expert knowledge, and might already be predisposed to participate in an AMR agreement. Countries in this group present the lowest risk if not initially included in an AMR agreement, because their antibiotic usage has decreased recently and few are major economic players.

Latvia (LVA) is an example of a Neutral Country that has been successful at addressing its own AMR issues but that may not be influential enough to drive participation in a global agreement. Locally, Latvia has focused on AMR in hospitals, including antibiotic prescription and administration practices.[38] Outside of hospitals, Latvia has one of the lowest rates of antimicrobial use among European Union (EU) countries.[39] Latvia's infection control legislation provides a good basis for improving infection control practices in healthcare.[39] Latvia's success can been used as an example for less wealthy countries to act on AMR; however, with low global influence, Latvia also represents a lower-priority country for recruitment into an AMR agreement.

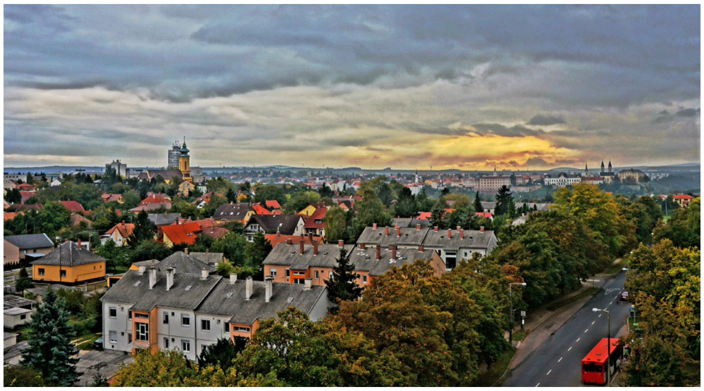

CC0 public domain photo by Cimabue

INFLUENTIAL STAKEHOLDERS

An important first step in analyzing the political feasibility of achieving global agreement on AMR is to identify key stakeholders who are likely to be affected by or play a role in AMR policies. At the international negotiation table, national representatives strive for agreements that promote common goals while remaining true to national priorities.[5] Domestically, key stakeholder groups influence national priorities by lobbying national governments to pursue policies favourable to their groups' interests.[4,5] Where tensions arise between key stakeholder interests and international policies, so can barriers to international agreement. Figure 3 lists the key stakeholders we identified, and summarizes the interests and tensions these stakeholders bring to an AMR agreement.

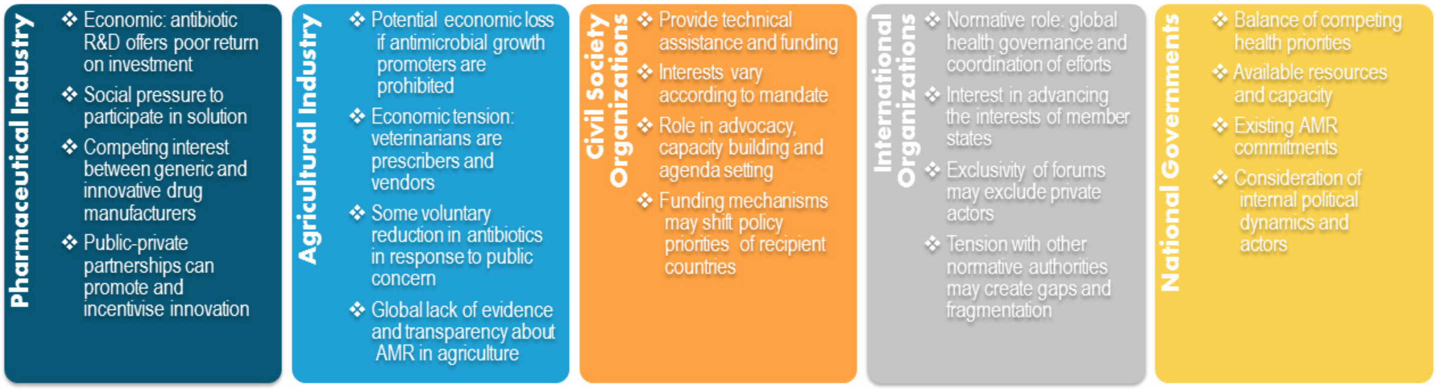

Figure 3 - Key Stakeholder Interests and Tensions

Pharmaceutical Industry

Stakeholders in the pharmaceutical industry include pharmaceutical innovators and generic drug manufacturers. Innovators are key to antimicrobial R&D, and both innovators and generic manufacturers can influence access to and conservation of antimicrobials through pricing, education, and responsible marketing.[40] Adding an estimated $1.23 trillion to the global economy annually, the pharmaceutical industry has strong lobby power to impact political interests and health policies such as those addressing AMR.[4,41]

The pharmaceutical industry's support for an international AMR agreement will likely depend on economic interests. Antimicrobial R&D offers a poor prospect of financial return.[7,42] Thus, pharmaceutical innovators are more likely to support an AMR agreement that provides appropriate financial incentives to invest in antimicrobial R&D. However, generic drug manufacturers will likely oppose R&D incentives or conservation measures that appear to restrict the generic antimicrobial market.[42] An international AMR agreement may face challenges in reconciling these competing interests.

The pharmaceutical industry has expressed interest in participating in the AMR solution. In January 2016, 85 pharmaceutical companies and nine industry associations representing both generics and innovators signed a declaration committing to act on AMR and calling on governments to collaborate.[43] Thus, the pharmaceutical industry is well-positioned to engage in discussions on an international AMR agreement.

Agricultural Industry

The agricultural industry relies heavily on antimicrobials, and is a key player in the global discussion on AMR. In 1997, an estimated 50% of all antimicrobials sold globally went to food animal production.[44] By 2030, livestock antibiotic use

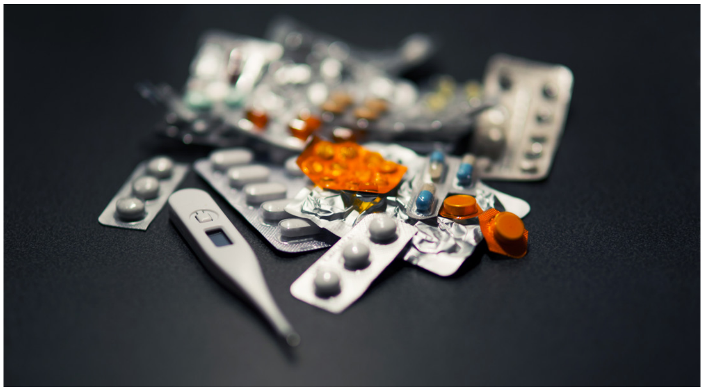

CC0 public domain photo by kaboompics.com

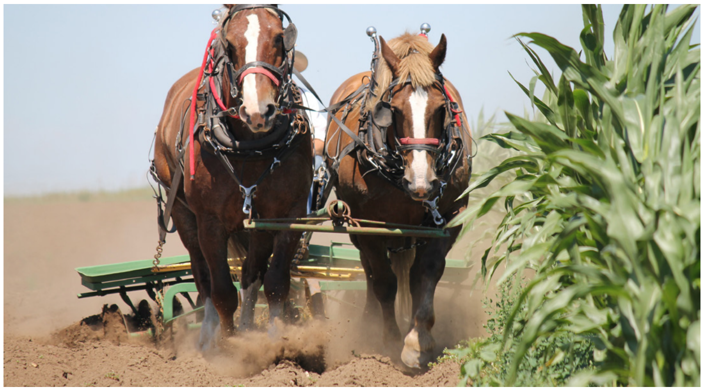

CC0 public domain photo by jmrockemand

is expected to increase by 67%, with agricultural use nearly doubling in the BRICS.[45] Using antibiotics of critical importance for human medicine to promote livestock growth and feed efficiency accelerates the development of drug-resistant human pathogens.[46,47]

The potential economic impact of an AMR agreement is a major concern for the agricultural industry. The WHO stated that banning antibiotic growth promoters (AGP) is one of the most effective AMR prevention measures.[48] However, there is a perception in the agricultural industry that AGP help maintain consumer confidence and allow food producers to meet growing global demands. Industry lobby groups argue that banning AGP creates financial loss and increases antibiotic consumption due to infections, illness, mortality, and animal suffering.[49,50] Thus, countries with a strong agricultural presence may face domestic pressures against conservation measures such as an AGP ban.

Some players in the agricultural industry have taken action to reduce antimicrobial consumption. For example, USA food providers such as McDonalds, Wal-Mart, and Costco have made efforts to raise antibiotic-free meat due to consumer demand.[51] Thus, governments could leverage this consumer demand to engage the industry in AMR discussions.

Civil Society Organizations (CSOs)

CSOs contribute to global health governance in many ways, making them important stakeholders in a potential international AMR agreement. For example, some CSOs advocate to change or improve existing policies or advance humanitarian issues, and can play a role in getting health issues on international political agendas.[52] CSOs can also promote surveillance and provide financial, material, and technical assistance to support health policy implementation.[52,53] Further, CSOs can provide oversight and facilitate capacity building in resource-poor countries with competing urgent health needs.[54-56]

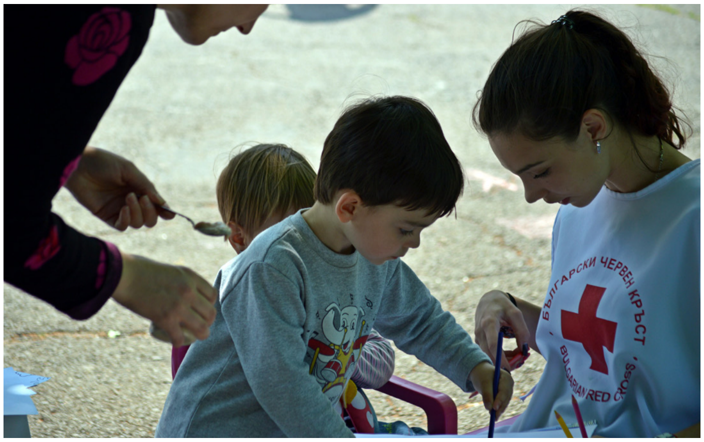

CC0 public domain photo by Div_Iv

CSOs can play a specific role in overcoming economic barriers to addressing AMR, and in turn gain financial leverage over health policy discussions.[53] Specifically, CSOs can help bridge gaps in wealth distribution around the globe by providing support to low- and middle-income countries (LMIC), which is essential to the success of global health policy.[10] Where their funding mechanisms require recipient countries to meet certain conditions before receiving funds, CSOs may influence a shift in national health policies as countries aim to meet funding requirements.[57]

International Organizations

International organizations such as the WHO are important actors in global health governance because they can coordinate efforts of other actors and stakeholders.[53] These organizations typically have normative or decisive mandates, and can provide a forum for and authority on specific matters to help establish international agreements. They can also form advocacy partnerships and engage stakeholders who may not otherwise have a voice in policymaking.[52]

International organizations are concerned with advancing the interests of their member states.[3] International organizations that propose health standards—such as the WHO—strive for widespread commitment to those standards. However, to establish global standards those organizations must find a position of leadership in an otherwise "crowded and often

chaotic global health architecture."[58] Further, member states exercise some control over international organizations' actions through voting mechanisms.[52] Acting in a manner that advances member state interests allows international organizations to garner confidence from those states and strengthens state commitment to proposed policies.

Domestic politics and private actor interests can influence the success or failure of international organization initiatives. Health is often seen as a national issue that requires nation-specific solutions.[55,59] Further, private actors such as industry and civil society can constrain an international organization's ability to set its own agenda or implement policies where those private actors have preferences for certain projects or agreements.[58,60,61]

National Governments

Ultimately, achieving a successful international agreement requires nations to come together at the negotiation table and commit to adopt and implement negotiated policies.[62] Economic and budgetary realities, health systems capacities and resources, and competing national priorities can either constrain or empower governments to act on global issues such as AMR.[63] States will also be concerned with satisfying domestic interests and pressures from major industry, civil society, and international organizations.

Internal political structure can also inform national participation in an international AMR agreement. Many federal nations divide powers over health with state or provincial governments, which can impact health policy implementation.[64,65] The USA, for example, filed a reservation to its commitment to the International Health Regulations (IHR), stating that the USA reserved the right to assume IHR obligations "in a manner consistent with its fundamental principles of federalism."[66] Further, the various interests of domestic ministries and government agencies involved in health policy could influence state positions in international negotiations. Where states perceive a risk that a particular nation's domestic realities or political structure will prevent that state from implementing negotiated policies, that nation may lose credibility and influence at the international negotiation table.[5]

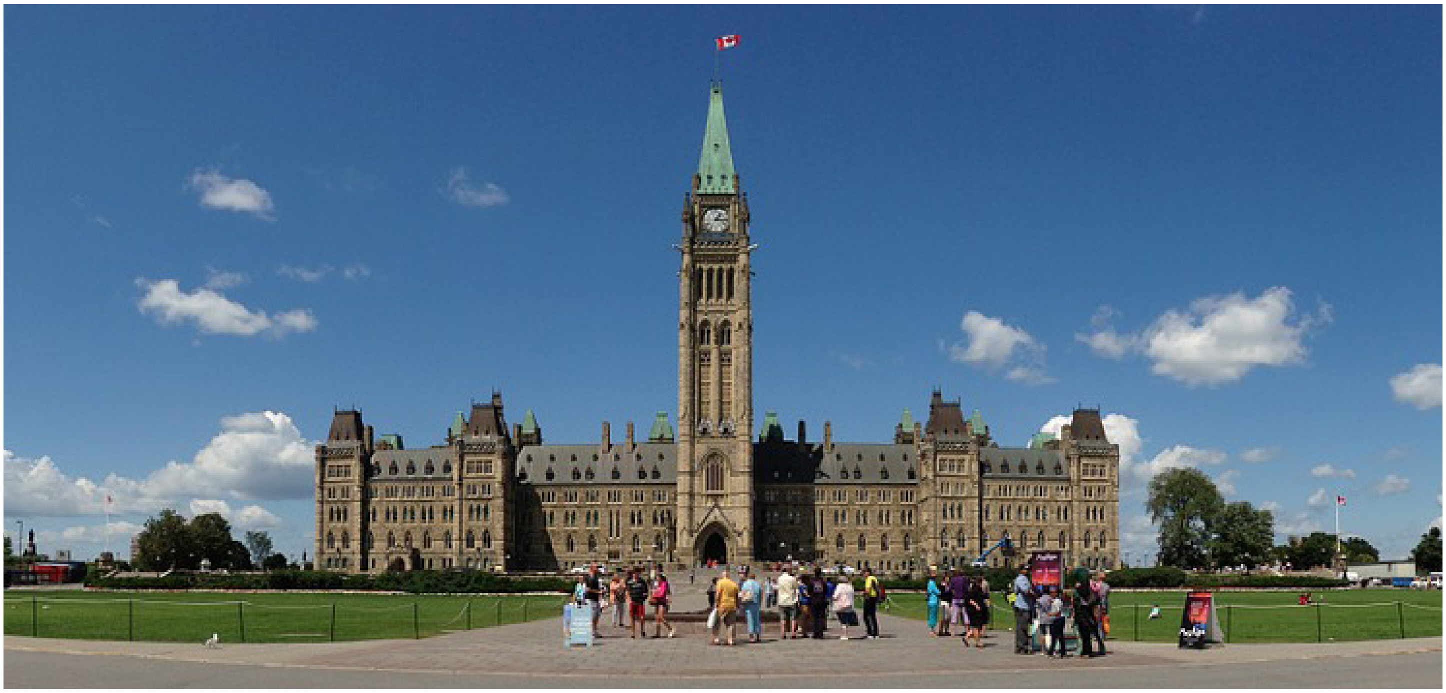

CC0 public domain photo by festivio

BARRIERS TO INTERNATIONAL AGREEMENT

Barriers to creating an international agreement on AMR

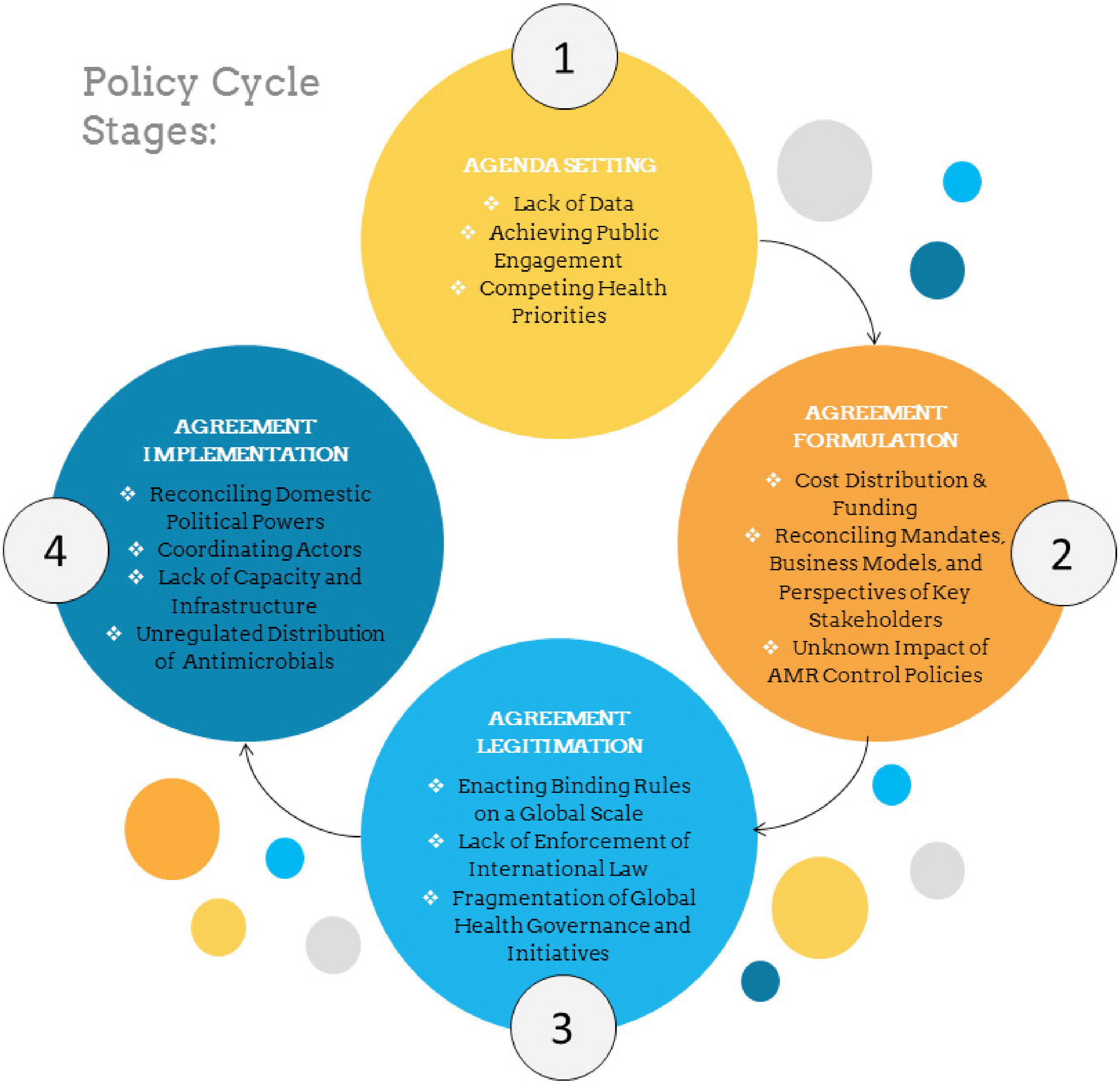

Figure 4. Identified Barriers to a Global AMR Agreement

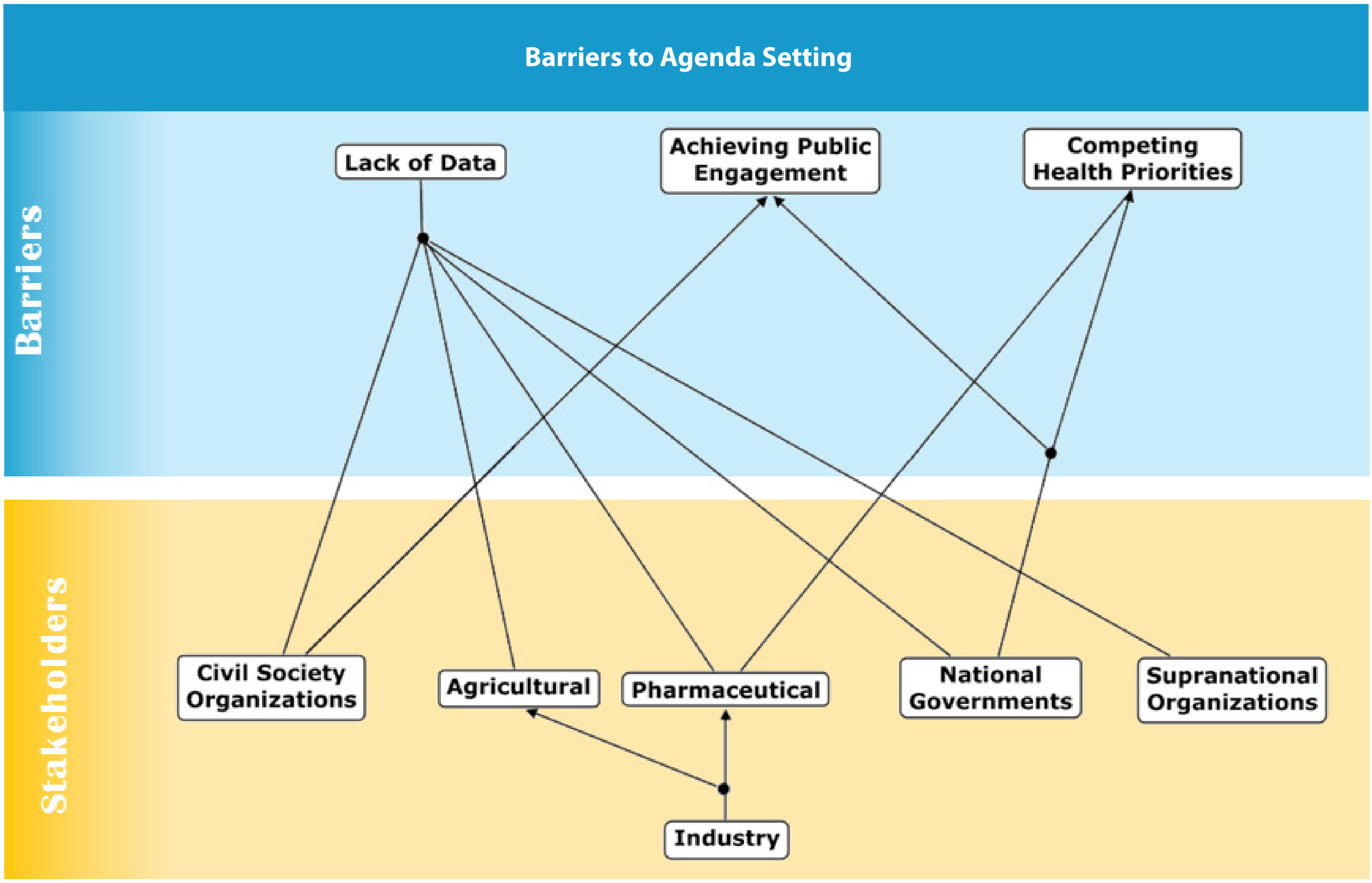

Figure 5. Stakeholder Interests and Barriers to Agenda Setting

1. Agenda Setting Barriers

Creating an international AMR agreement requires consistent public and political engagement with the AMR issue. While political goals are often short-term due to election cycles,[34] AMR is a long-term battle that requires long-term funding. Public engagement with health issues can influence the success of health policies,[4] and the WHO has recognized that public awareness of AMR is low.[67] Thus, increasing awareness through civil society efforts and educational campaigns, such as the WHO's first World Antibiotic Awareness Week in November 2015,[68] may help put AMR on national health policy agendas.

Competing health priorities may create barriers to keeping AMR front and centre on political policy agendas. Historically, short-term emergencies such as the 2003 SARS outbreak have pushed AMR to the background.[67] Further, governments must find ways to include AMR in national health policy agendas along with local, longer-term priorities such as HIV/AIDS, malaria, or chronic diseases, despite limited resources.

Lack of data is an ongoing barrier to putting AMR on policy agendas. To measure the magnitude and scope of the problem, countries need adequate surveillance of AMR in humans, animals, and of antibiotic sales and prescribing practices.[69] However, many countries have little or no access to comprehensive data.[67,69,70] International discord on surveillance practices makes producing reliable AMR data even more difficult.[69] Uncertainty as to what constitutes therapeutic or nontherapeutic antimicrobial use in livestock makes it difficult to assess the extent to

which these practices exist in agriculture.[46] Economic and policy evaluations as well as intervention research would provide decision makers with better evidence to support policy decisions.

2. Agreement Formulation Barriers

A lack of data may also hinder AMR policy formulation. To date, there have been insufficient efforts to evaluate the impact of existing AMR control policies, which creates a major practical challenge to crafting and estimating the effect of new policies. Larger-scale evaluations or comparative effectiveness studies (e.g., for best farming practices)[71] would help determine the most effective provisions to include in an international agreement. Many of the evaluations that currently exist are single hospital interventions, which are typically analyzed only for economic impact at the hospital level.[72] Securing funding for international AMR policies may also constrain policy formulation, as policymakers may face pressures to focus on particular issues to secure funding. For example, where CSOs place conditions on grants, policies may shift to meet those conditions.[57]

Reconciling key stakeholder mandates, business models, interests, and perceptions presents a further challenge. Different agencies have different perspectives on key AMR policies. Where high-power, organized groups expect to bear most of the cost of health policies, policymakers may face strong opposition.[4] For example, the agricultural industry will not likely support an international AMR agreement without cost-effective alternatives to antimicrobials.[71] Pharmaceutical innovators are unlikely to support an international AMR agreement without incentives to invest in R&D. However, incentives cannot be achieved by increasing prices or sales volume, as such measures would undermine access and conservation,[11,40] and could be opposed by civil society. Thus, alternative funding models are needed to support

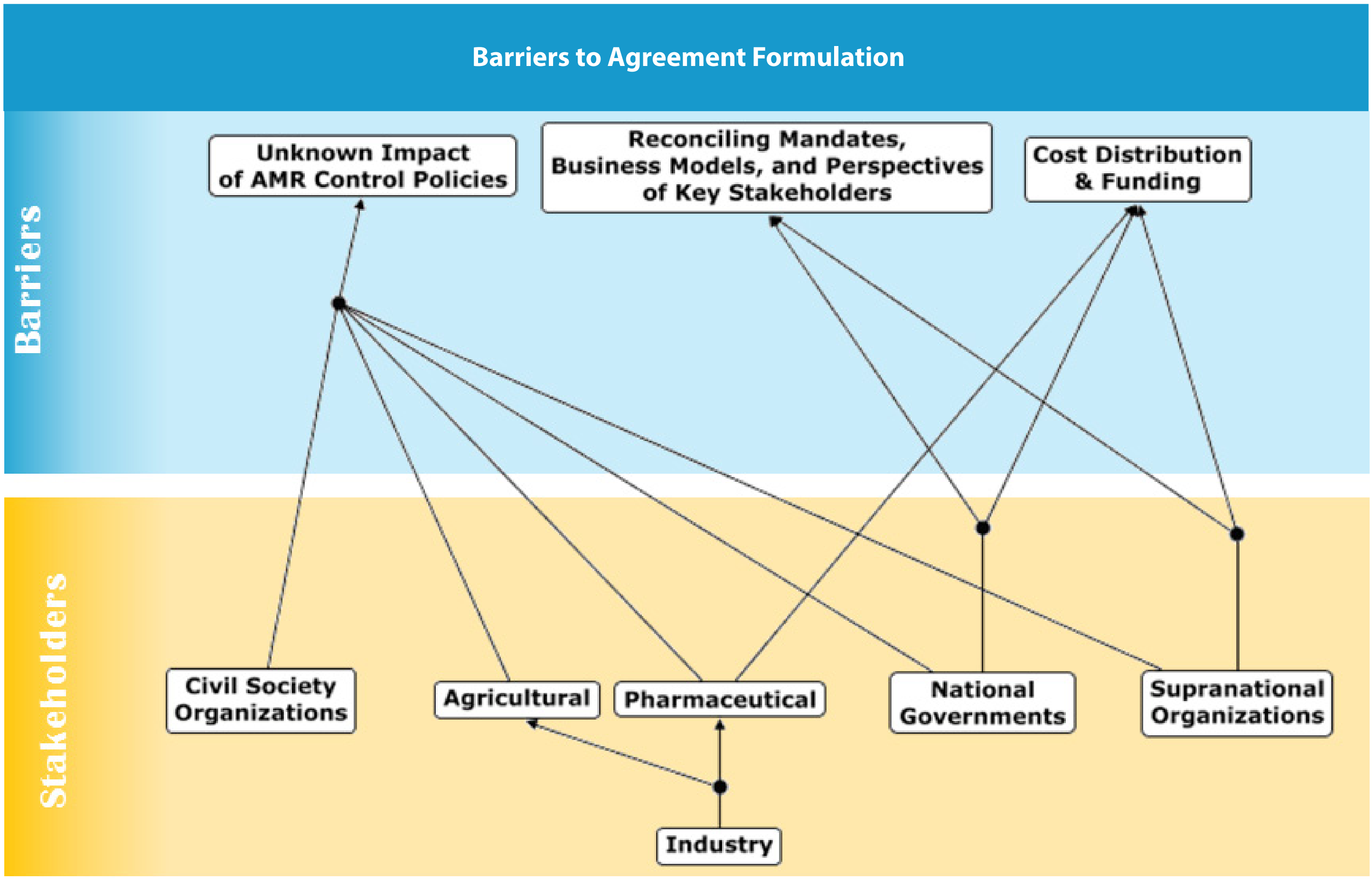

Figure 6. Stakeholder Interests and Barriers to Agreement Formulation

antimicrobial R&D.[73,74] However, countries with a large generic pharmaceutical presence, such as India, might fear that such an agreement could shut generic manufacturers out of the global market.[42,75] Formatting a single agreement to take these perspectives into consideration could be challenging.

Similarly, reconciling complex international regulatory standards in a way that encourages stakeholders to participate may be challenging. For example, the typically complicated and expensive regulatory requirements for developing new antimicrobials currently acts as a deterrent to innovation.[40]

3. Agreement Legitimation Barriers

Garnering legal support for an international AMR agreement presents further challenges. A legally binding and enforceable agreement could ensure certain AMR policies are adopted and implemented on a global scale.[76] However, few entities have the authority and capacity to enact binding rules on a potentially global scale. Choice of forum can determine which actors can be directly engaged. In global health governance, the WHO is the most likely forum of choice given its unprecedented constitutional mandate.[58] However, with the emergence of new fora and shifts in power, global health governance has become fragmented over the years,[77] possibly presenting challenges in the creation of global normative agreements.

International law can also be difficult to enforce. For example, countries can ratify legally binding agreements while opting-out of particular commitments or submitting reservations.[78] Addressing AMR at a global level will require mechanisms for achieving widespread implementation, compliance, and accountability.[79]

Fragmentation of existing AMR efforts may also create barriers to policy legitimation. Some transnational entities

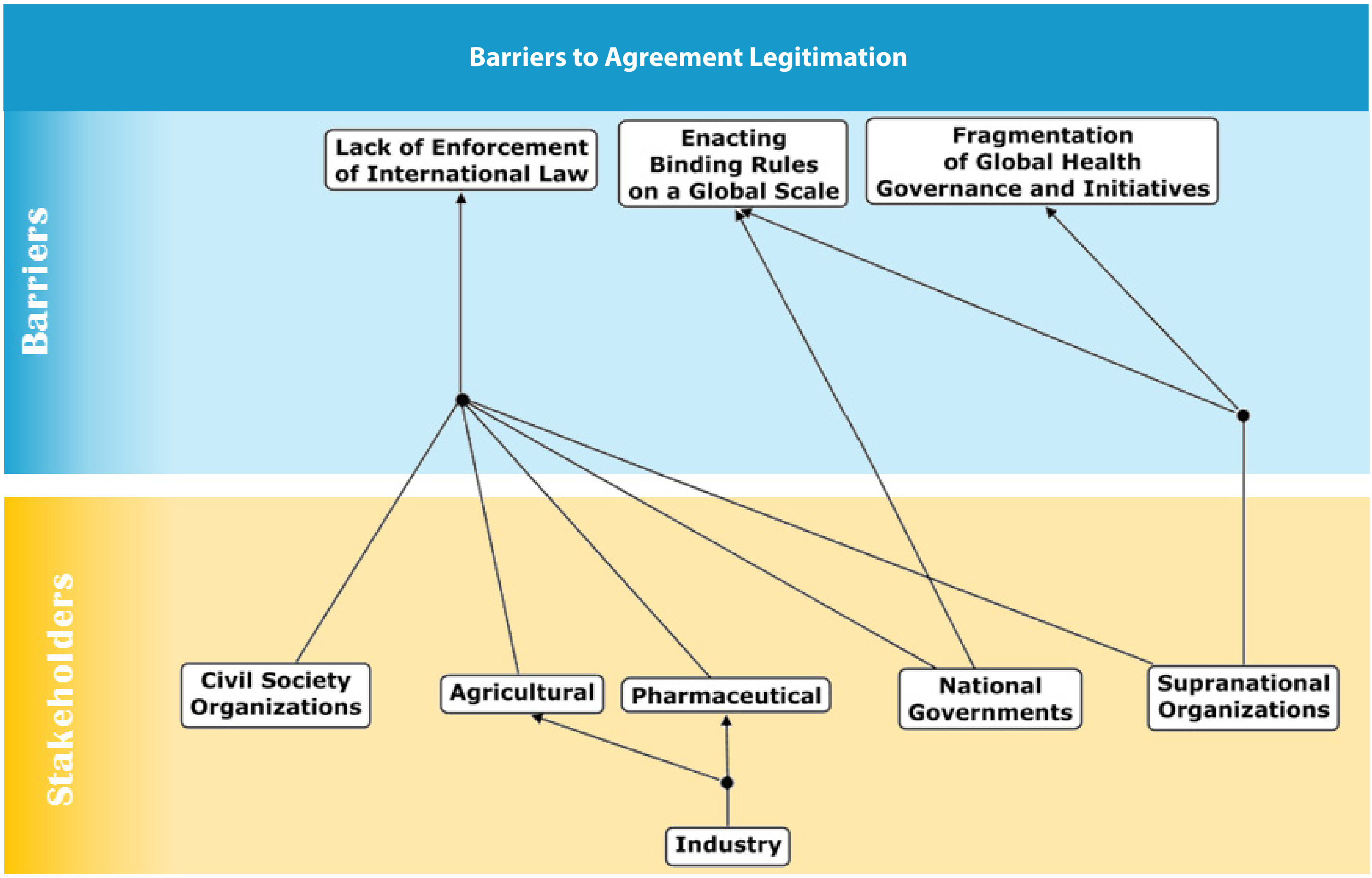

Figure 7. Stakeholder Interests and Barriers to Agreement Legitimation

have proposed norms and standards to respond to AMR, and individual countries have started developing and implementing national strategies. The WHO Global Action Plan urges countries to develop national strategies that comply with international standards such as the Codex Alimentarius Commission, Food and Agriculture Organization of the United Nations, and World Organisation for Animal Health to ensure a "one-health" approach.[74] Any new agreement will likely face challenges in harmonizing existing efforts and frameworks while addressing national and regional participation and needs.[80]

4. Agreement Implementation Barriers

To implement an international AMR agreement, actors and stakeholders will need to continue to cooperate at national and international levels. As with policy formulation, the challenge will be to reconcile stakeholder interests as well as international regulatory standards in a manner that permits sustainable commitment to AMR policy. Similarly, countries may face challenges reconciling domestic powers to implement international standards, particularly in federal countries where powers over health are divided with domestic states or provinces.[64,65] Successful implementation will also require enforcement and accountability mechanisms.[79]

Weak infrastructure and capacity in research, surveillance, manufacturing, sanitation, and infection control may also hinder policy implementation. For example, research infrastructure is lacking for antimicrobial R&D due to poor financial incentives and past perceptions that research was no longer needed in this field.[40] Further, many countries lack the capacity to enforce laws against counterfeit pharmaceuticals.[81] At the most basic level, sanitation and infection control remain a problem in many places, and antibiotics are used as a stopgap to ensure patient safety.[70]

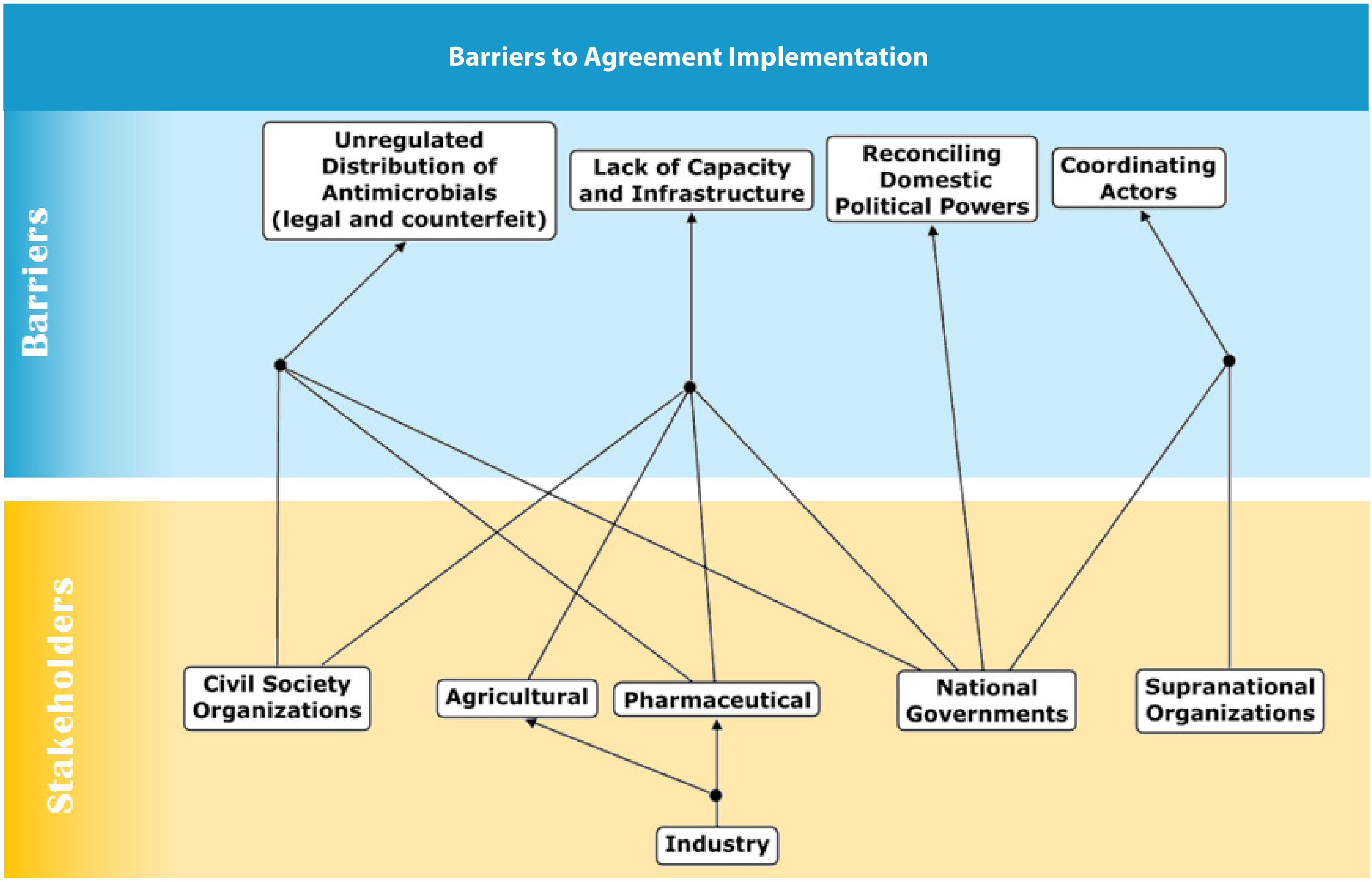

Figure 8. Stakeholder Interests and Barriers to Agreement Implementation

POLITICAL FEASIBILITY ANALYSIS

To effectively combat AMR, global action needs to address three key areas simultaneously: access, conservation, and innovation. Solving each of the access, conservation, and innovation problems will engage a different combination of stakeholders and interests, and each problem faces a different set of barriers in the policymaking cycle. Thus, the political feasibility of achieving coordinated global action on AMR depends on the likelihood that key actors can be engaged to overcome barriers to achieving global policies on access, conservation, and innovation.

Achieving Agreement on Access

Despite the growing problem of AMR, inadequate access to antibiotics still claims far more lives than antibiotic resistance.[82] A lack of infrastructure, financial constraints, and human resource limitations[83] present major barriers to achieving global agreement on improving access, mainly at the agenda setting and implementation stages of policymaking. Engaging Initiator and select Pivotal Countries will facilitate solving the access problem for Pivotal and Follower Countries by helping to overcome resource limitations.

Summary: Access

Key barriers to overcome: (1) lack of infrastructure, (2) financial constraints, and (3) human resource limitations.

Key actors and roles: Initiator Countries, as well as a few select Pivotal Countries, must be engaged to provide resources and build capacity to overcome barriers in Follower and Pivotal Countries that struggle with inadequate access to antimicrobials.

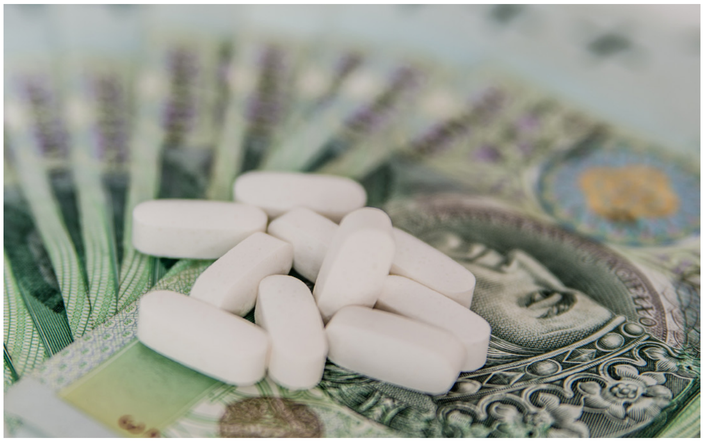

CC0 public domain photo by jarmoluk

Overcoming Resource and Capacity Limitations with Leadership from Targeted Countries

Pivotal and Follower Countries both struggle with access problems, but often as a result of different contributing factors. To improve access to antimicrobials, any proposed agreement will need to account for the complex background against which access issues arise in each country. For example, India has a high GDP and has a substantial generic pharmaceutical market,[84] yet it still struggles greatly with access. Thus, select Pivotal Countries such as India, despite having access problems, could contribute to the solution.

To address resource limitations, Initiator Countries could be engaged to provide financial support for access initiatives. Some of these countries—such as the USA, the United Kingdom and Germany—are already top international aid contributors,[85] and will therefore likely be willing to provide financial assistance. Initiator Countries will likely be even more willing to contribute if the financial burden is fairly distributed among participating countries through, for example, a proportional pooled global fund. Engaging CSOs to provide support for funding initiatives could further reduce the financial burden and bolster Initiator Country willingness to participate.

It has been suggested that to overcome resource limitations to addressing AMR it will be necessary to move away from the "donor model" in favour of a more sustainable arrangement.[83] As tensions can arise between CSO mandates and broader public health priorities,[57,60] funding mechanisms such as a pooled fund between national governments could help ensure greater control over the focus of efforts during implementation.

Strategies aimed at improving access as well as infrastructure and capacity would likely gain more traction among Pivotal, Follower, and even Neutral Countries with competing health priorities, as such measures would address more than one health need. For example, strengthened public health systems can reduce use of antimicrobials as a stopgap measure,[82] and improved sanitation can reduce disease burden, which is highest per capita in Follower Countries.[86] A multi-targeted approach is more sustainable than simple funding mechanisms because it addresses access issues at their core, which is likely to appeal to countries facing complex barriers to access.

Achieving agreement on access is also more likely to be feasible if measures are tailored to local needs and capacities. For example, countries with emerging pharmaceutical markets tend to implement policies that support local drug manufacturers.[87] These countries are therefore likely to oppose, or face barriers to implementing,[88] strict blanket regulatory frameworks. Policies that provide countries with some flexibility to account for local contexts will likely receive broader support.

The high costs associated with certain medicines creates an obstacle to accessing those medicines in many countries. A core group that includes countries such as India—a Pivotal Country with a large stake in the generics market—could be engaged to increase access to affordable generic medications in resource-poor countries. Still, efforts to engage the generic pharmaceutical industry might see limited success in addressing the access problem, because many Follower Countries are reluctant to use generics, in part due to product quality concerns that lead to distrust of off-brand medication.[83] Improving capacity and strengthening regulatory systems among generic manufacturers could help dispel these fears.[83,88]

Tensions could arise within the pharmaceutical industry if branded products lose part of their value-added as a result of the influx of quality-assured generics on local markets and have to lower their prices to remain competitive. Drug manufacturers have been known to discontinue drugs in unprofitable markets,[89] which could impact local economies. Any proposed agreement will need to be adjusted to local market realities and may benefit from engaging participants early on through strategic partnerships with CSOs, existing local entities, and governments to facilitate market access and support more predictable returns.[87]

Finally, it is important to note that any agreement that includes measures that impede on countries' authority over health matters will likely be met with great reluctance. However, the value-added of a global agreement on AMR that facilitates and coordinates efforts can be leveraged to increase interest. This could create an important incentive for countries with infrastructure limitations—such as many of the Follower Countries—to participate in an international agreement on AMR. International entities in particular could help in this respect.[90]

Achieving Agreement on Conservation

Misuse and overuse of antimicrobials drives resistance. A commitment to antimicrobial conservation can ensure that these drugs remain useful for longer. However, data gaps and poor regulation of antimicrobials create major barriers to agreement on conservation at the agenda setting and policy implementation stages. While antimicrobial regulation can be addressed by a small group of invested Initiator and Pivotal Countries, surveillance is a broader conservation measure that requires global commitment and coordination.

Summary: Conservation

Key barriers: (1) lack of data on AMR, and (2) global disparities in regulatory frameworks.

Key actors and roles: While surveillance efforts must be undertaken on a global scale to be effective, engaging key Initiator and Pivotal Countries to strengthen regulatory frameworks on human and animal use of antimicrobials could greatly improve stewardship worldwide.

Global Commitment to Overcoming Data Gaps

Lack of data on AMR in both humans and animals presents a major barrier to international action on conservation, because complete global data is necessary to track and prevent the spread of resistant infections and to improve the accuracy of patient diagnoses. Creating a global surveillance network faces two further barriers: a formulation-stage barrier of perceived repercussions of global data-sharing, and an implementation-stage barrier of lack of capacity to undertake surveillance.

A core group of Initiator and Pivotal Countries can be expected to lead efforts to develop global surveillance capacity. These are countries that have taken steps to combat AMR, and require global surveillance data to ensure that AMR imported via travel and trade does not reverse the work they have done. As such, Initiator Countries and some Pivotal Countries—such as the Netherlands and Denmark—have already allocated significant resources to surveillance at the national level.[91,92] Some Initiator Countries, including the UK and Norway, have also shown commitment to increased global collaboration on AMR in their National Action Plans.[93,94] To ensure continued success, these Initiator Countries will need to convince their major trading partners among the Pivotal Countries to apply their own resources to surveillance efforts for their mutual benefit.

Other Pivotal Countries have not yet substantially engaged in AMR efforts, though many—such as the BRICS—would likely have the financial resources to do so if they perceived a trade benefit to participating in surveillance and data-sharing. Pivotal Countries that are major world exporters could be reluctant to share data if they perceive a threat to their ability to conduct international trade.[95] For example, banning food exports from countries with high antimicrobial consumption rates could act as a disincentive for certain producers to share data due to fears of being excluded from the global market. On the other hand, if data-sharing is required to access certain markets, countries might be incentivized to make this information available. Still, Pivotal Countries will not want to participate in surveillance and data-sharing if they perceive that doing so will create an undue burden. Engaging other country types to share this perceived burden could alleviate concerns and facilitate action amongst Pivotal Countries.

Follower Countries do not currently have the resources to undertake surveillance. With competing political, economic, and health priorities, Follower Countries are not naturally disposed to dedicate resources to AMR. It will be important to convince these countries that their participation is vital to prevent gaps in surveillance data. To attract Follower Countries, Initiator Countries need to develop mechanisms to fund infrastructure development and consider tying these supports to other local health needs.

Global participation is key for any agreement that addresses surveillance. Incomplete data is less useful to agreement participants, which reduces the advantages of taking part in surveillance efforts. However, improved surveillance is necessary to facilitate further action on antimicrobial access, conservation, and innovation. Thus, obtaining global agreement on surveillance measures should be a priority in the AMR response.

Targeted Commitment to Overcoming a Lack of Regulation

Global gaps in antimicrobial regulations, in both human and animal contexts, present a major barrier to implementing conservation efforts.[96] Harmonized international regulations or policies, including guidelines and action plans, are integral to an agreement that addresses conservation. Such regulations might address, for example, sales practices in humans or the agricultural use of AGPs. However, regulatory measures must be sensitive to the interests and capabilities of target countries. Industrialized Initiator Countries will need to be engaged to provide financial, technical, and legal expertise to support participation by developing Pivotal Countries.[64]

In humans, non-existent, inadequate, or unenforced regulations on prescribing and sales practices contribute to the spread of AMR, providing a key target for regulatory measures. Enforceable regulations of OTC sales practices, for example, could encourage prudent use of antimicrobials and eliminate financial incentives to overprescribe. A core group of Initiator and Pivotal Countries ought to take the lead on regulatory measures related to OTC sales practices, as many Initiator and some Pivotal Countries have already taken steps to address this problem.[14,97,98]

Many Follower Countries are not currently in a position to engage in an agreement designed to regulate OTC sales. Many Follower Countries lack legislation and enforcement mechanisms. Furthermore, lack of point-of-care access means that these countries are likely to face substantial barriers to implementing OTC regulations without compromising access to antimicrobials. Thus, Follower Countries will likely require support and guidance from Pivotal and Initiator Countries to take action.

Developing Pivotal Countries could present some opposition to becoming involved in OTC regulation measures. For example, Pivotal Countries with strong interests in the pharmaceutical industry will likely oppose OTC regulations if they perceive a risk of economic losses. Pharmaceutical industry opposition to regulating OTC practices in Mexico[96]—an Initiator Country—foreshadows difficulties that countries may face in implementing OTC regulations.

In spite of potential stakeholder opposition, an international agreement that addresses OTC sales regulation should include Pivotal Countries that have not yet addressed this issue to support future participation in Follower Countries. If a core group of Initiator Countries and Pivotal Countries takes steps to regulate antimicrobials—such as the BRICS—this could influence additional Pivotal Countries to participate at the initial stages and facilitate action to conserve antimicrobials.

In the animal sector, the use of growth promoters is one of the most controversial practices contributing to AMR rates. Since AGP use is controversial, global agreement could be hampered during attempts to reconcile the diverse interests of countries and stakeholders. A more feasible approach to addressing AGP use would be to engage a smaller group of Initiator and Pivotal Countries with strong agricultural industries. The growing recognition among some Pivotal Countries—such as the BRICS—of AMR in the agricultural industry means these players would be best positioned to act on AGPs alongside Initiator Countries, some of which have already taken the lead on the issue.[22]

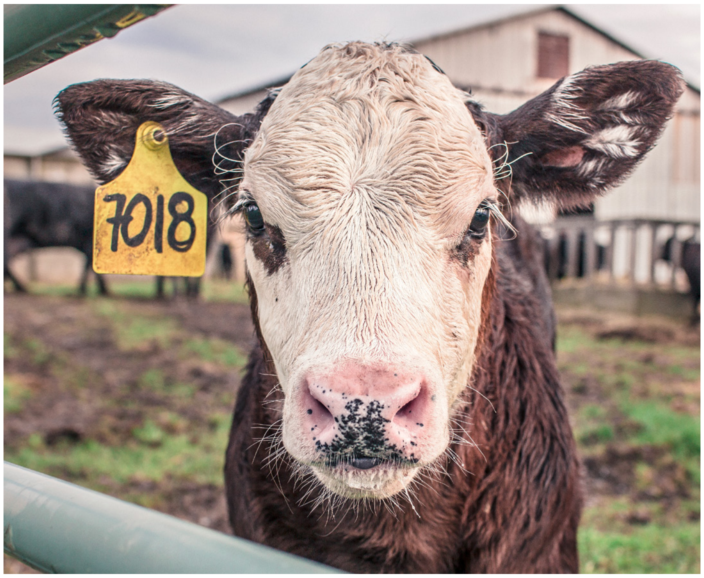

CC0 public domain by Ryan McGuire

Initiator and Pivotal Countries with economic interests in the agricultural industry may face pressures oppose agricultural conservation measures such as banning AGPs. The agricultural industry is generally against banning AGPs because of its interests in maintaining efficiency and profitability.[49,50] Industry resistance would likely be particularly prominent in rapidly expanding Pivotal Countries, which need to provide ever-increasing amounts of food. However, some large industry players in Initiator Countries have expressed a willingness to address the issue, so engaging these Initiator Countries may be less of a challenge. The veterinary profession may also oppose conservation measures in animals, depending on whether national regulations permit veterinarians to profit from both prescribing and selling antimicrobials.[99] However, veterinary opposition could be minimal in practice.[100]

Including policies that promote alternatives to AGPs as part of proposed conservation measures could facilitate agreement from Initiator and Pivotal Countries with major agricultural industries. Such measures could include new feeds, improved housing, and infection control.[101,102] Further, developing new formulations of old antimicrobials could minimize the use of critically important antimicrobials in animals.[103] Alternatives must however be practical and affordable, especially in developing Pivotal Countries.[71,104]

Agreement on regulatory measures to promote conservation is more likely to be politically feasible if policies are tailored to local realities,[11,105] given the challenges associated with attempting to implement harmonized regulations across country types. Some Pivotal and Follower Countries may not have the capacity to implement advanced regulatory policies, meaning that it may not be feasible for these countries to adopt the same regulations as each other or Initiator Countries. On the other hand, Initiator Countries may face problems adapting entrenched regulations to recognize, for example, alternatives to antimicrobials.[106] Thus, regulatory policies promoting conservation of antimicrobials must be tailored to local capacities and realities to achieve agreement across country types.

Achieving Agreement on Innovation

Summary: Innovation

Key barriers: (1) reconciling key pharmaceutical stakeholder interests, (2) distributing costs to spur innovation, and (3) lack of capacity and research infrastructure.

Key actors and roles: Initiator countries must take the lead on innovation, but Pivotal Country engagement can both help incentivize innovation through sharing of costs and reconcile potentially opposing interests.

The global AMR response must provide financial incentives to stimulate continued antimicrobial innovation without compromising access or conservation through sales volume or pricing.[7,73,74] Achieving sustained antimicrobial R&D will require policymakers to reconcile key stakeholder interests and cost distribution concerns at the policy formulation stage, and overcome capacity and infrastructure barriers at the implementation stage. While Initiator Countries can take the lead in solving the global innovation problem, leveraging support from other country types—particularly Pivotal Countries with emerging pharmaceutical markets—could bolster the political feasibility of creating new incentives for innovation.

Overcoming Capacity and Infrastructure Barriers with Initiator Countries

Initiator Countries with high interests and investments in pharmaceutical R&D should take the lead on antimicrobial innovation.[107] In fact, select Initiator Countries could tackle antimicrobial innovation alone.[76] High-income countries hold most of the world's innovation capabilities,[83,108] and several Initiator Countries—such as the USA, Japan, and

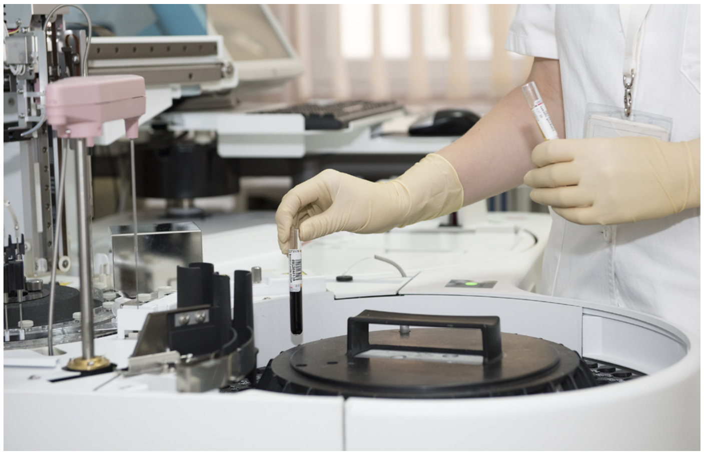

CC0 public domain photo by DarkoStojanovic

France—invest heavily in pharmaceutical R&D.[109] The USA has also identified investment in antimicrobial R&D as a key priority in its national strategy on AMR.[110] Initiator Countries are therefore best positioned to overcome any capacity or research infrastructure barriers associated with kick-starting the antimicrobial pipeline.[40]

Reconciling Key Stakeholder Interests in Initiator and Pivotal Countries

Supporting pharmaceutical R&D sector interests will be a key concern for Initiator Countries with strong pharmaceutical markets in taking the lead on antimicrobial innovation. Initiator Countries include most of the top eight developed pharmaceutical markets, where the pharmaceutical R&D sector accounts for nearly 75% of the market share and the provides the largest economic value-added of the pharmaceutical industry.[84,111] Further, industry organizations can provide a powerful platform for members of the R&D sector to lobby national governments for health policies favouring R&D sector interests.[4,112] In light of recent interest from the industry,[43] an innovation agreement that provides a mechanism for fair and sustainable compensation for antimicrobial R&D will likely see little resistance from the R&D sector.

Initiator Countries could receive opposition from the generic pharmaceutical industry if proposed strategies to incentivise innovation appear to limit the generic antimicrobial market in those countries. For example, generic manufacturers have opposed extending patent rights for innovators.[42] The global generic pharmaceutical industry is growing,[108,109] and like pharmaceutical innovators, generic manufacturers can leverage powerful industry groups to lobby governments to protect their interests.[42] However, given the relative strength of the R&D sector in many Initiator Countries, and the expressed interests within these countries—including from generic manufacturers[43]—in supporting antimicrobial innovation, generic opposition is unlikely to prevent key Initiator Countries from taking the lead on the global innovation response.

Pivotal Countries with emerging pharmaceutical markets could be engaged to support global antimicrobial innovation efforts. The four fastest growing pharmaceutical markets—China, Brazil, Russia, and India[84]—are all Pivotal Countries, and antibiotics represent the second largest drug class for spending in these markets.[113] Thus, these countries present promising targets for supporting sustained, global antimicrobial innovation initiatives.[107]

Pivotal Countries will likely resist supporting an innovation agreement unless that agreement includes protection for the generic pharmaceutical industry. Generic pharmaceuticals account for nearly two-thirds of the market share in emerging pharmaceutical countries, due in part to a need for cost-controlled drugs and a tendency to favour local manufacturers, which often fall in the generic sector.[84,111] Countries with a large generic pharmaceutical presence, such as India, may resist stewardship measures perceived to exclude the country's generic manufacturers from the global market.[75] Similarly, these countries will likely oppose an innovation agreement perceived to be unfavourable to the generic industry. Protecting generic pharmaceutical interests through, for example, engaging generic manufacturers in these countries to provide priority access to novel antimicrobials in low-income markets,[73] could facilitate support from Pivotal Countries.

Global Commitment to Overcoming Cost Distribution and Funding Barriers

While select Initiator Countries could solve the innovation problem alone,[76] an innovation agreement could leverage support from a broader range of countries to overcome policy formulation barriers of cost distribution and funding. Key Initiator Countries are more likely to resist leading the innovation response if they perceive they will bear an unfair cost burden.[4] If new antimicrobials must be viewed as a "global public good", then all countries who benefit should contribute to creating that public good.[73] An agreement requiring all countries to contribute proportionally to funding for innovation proportionally could bolster Initiator Country support.

Pivotal, Follower, and Neutral Countries with competing priorities and minimal pharmaceutical markets would likely resist investing in R&D. However, including these countries in the innovation solution could help facilitate broader access

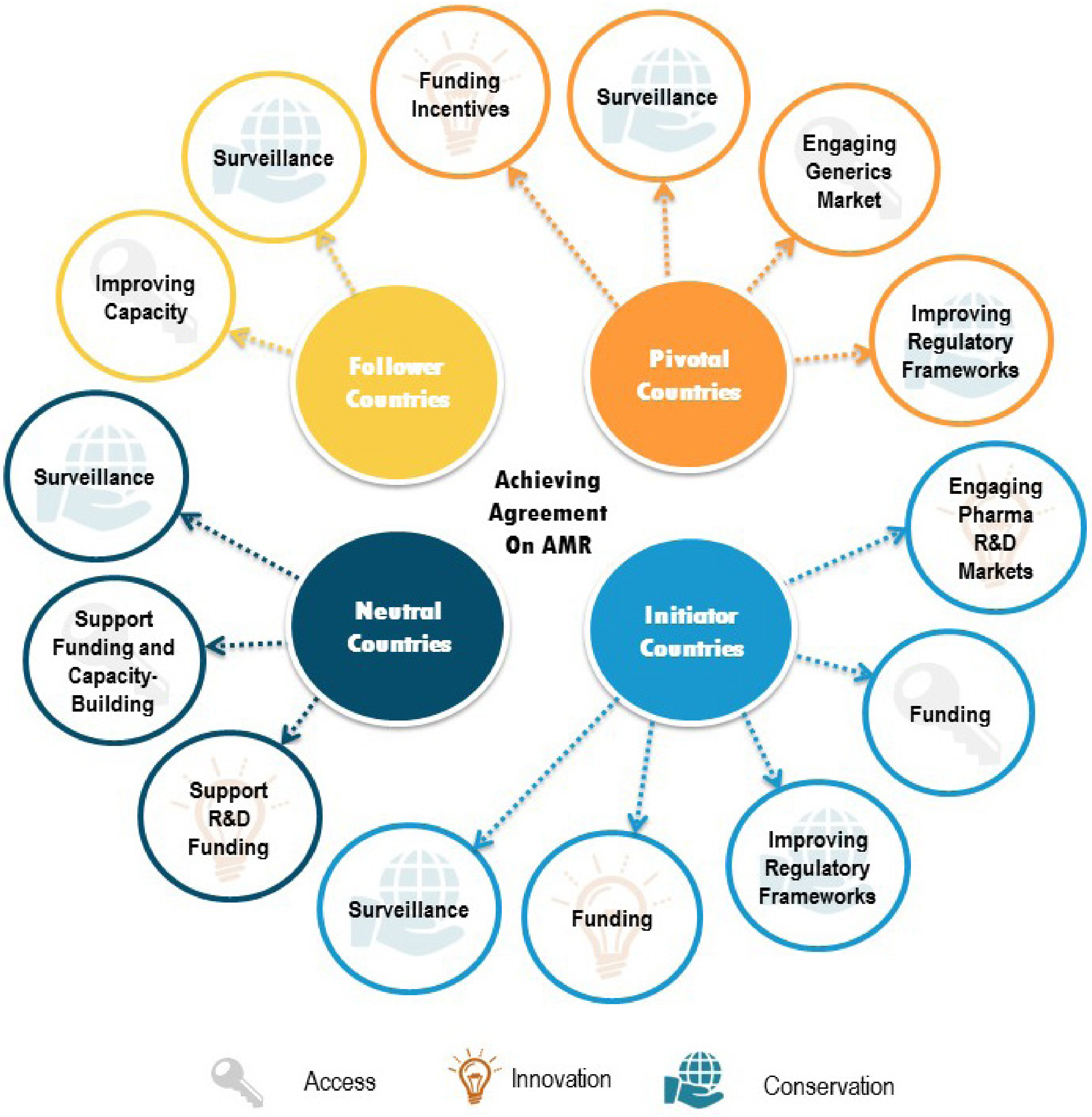

Figure 9. Pictograms from The Noun Project, created by Egor Culcea, Icon 54, and Korawan.M

and conservation measures.[76] For example, lower-income countries could be offered special access to new antimicrobials in exchange for contributing to a global innovation fund and implementing conservation policies.[76] Still, competing priorities and resource concerns likely make engaging all countries to support antimicrobial innovation less feasible than engaging Initiator Countries to take the lead.

Achieving Agreement on AMR

Politically Feasible Action on AMR Starts with Initiator and Pivotal Countries

9Global coordinated action addressing access, conservation, and innovation of antimicrobials is necessary to combat AMR. However, a global-scale AMR agreement on access, conservation, and innovation is not immediately politically feasible without additional incentives or supports. For many countries, including many Pivotal and Follower Countries, a lack of resources and capacity, competing domestic priorities, and perceived threats to economic growth present major barriers to getting AMR on the political agenda, let alone formulating or implementing AMR policies. In addition, fragmentation of global health governance creates additional challenges to legitimizing an actionable global-scale agreement on AMR. Thus, global action on AMR will need to begin with a select subset of countries.

Despite difficulties in achieving global collective AMR action, there is one realm in which immediate and global agreement is essential to initiate a coordinated response: surveillance of AMR and antimicrobial consumption in both humans and animals. A lack of global data on AMR is one of the biggest barriers to agenda setting across all other areas on access, conservation, and innovation. An agreement on surveillance, though still difficult to achieve, has a narrower focus that may be more conducive to finding common ground. A surveillance agreement also has specific outcomes that are more amenable to using enforceable mechanisms. Agreement on this key issue will provide more evidence to persuade Pivotal and Follower Countries of the need for action on other components of AMR. Beyond initiating surveillance measures, our analysis suggests that to achieve coordinated global action on AMR, we must find leaders among Initiator and Pivotal Countries who can initiate agreement on access, conservation, and innovation measures. Initiator Countries were identified as key players in each of the access, conservation, and innovation components of our analysis. Yet without engaging Pivotal Countries, who also have substantial global influence, an agreement will likely receive little traction on a global scale and fail to lead to real global change. Some of these Pivotal Countries are already identified supporters, while others may need information, support, or incentives before they see the value in participation.

Leveraging smaller groups of specialized Initiator and Pivotal Countries to drive agreement on AMR is most politically feasible, particularly at the agenda setting and policy formulation stages, because fewer countries means reconciling fewer interests and making fewer trade-offs. Bearing in mind the eventual need to attract participation from many nations and to widely implement policies, some agreements may be best served by resolving differences among the small group of countries. For example, conservation in agriculture could first be addressed among countries with the largest agricultural export sectors. Having achieved a measure of consensus among this group, the agreement could then be modified to meet the needs of additional countries.

To retain the interest of Follower Countries and encourage their eventual integration into a proposed agreement, countries that need to build capacity before participating in an agreement, or that require support from Initiator or Pivotal Countries, could be engaged as observers. As observers, these countries would be able to participate in policy discussions without needing to make commitments they could not feasibly implement.[106] Allowing Follower Countries the flexibility to engage internationally on AMR without immediate financial or resource commitments could support their eventual participation and gradual integration into an agreement.

Broader coordinated action can be increased using incentives

and trade-offs to gain agreement from countries outside the core group of leading countries. For example, Pivotal Countries with an emerging pharmaceutical market could be engaged to support access to affordable antimicrobials in resource-poor regions in exchange for contributing to funding for innovation. This type of measure would alleviate the financial burden placed on Initiator Countries who are driving innovation and facilitate access in lower-income countries—even if they are not part of an initial AMR agreement. Alternatively, lower-income Pivotal Countries could be offered access to research funding and new innovations in exchange for adopting and implementing conservation policies, which could facilitate agreement on innovation and conservation.[76]

Relying on certain Initiator and Pivotal Countries to initiate a global solution to AMR places some pressure on those countries from a resource perspective. However, the potential benefits of taking the lead outweigh the costs. Because AMR is a global issue, each country stands to benefit when other countries take action. If high-influence countries can initiate global AMR policies, Follower and Neutral Countries will be easier to attract. Each country has traditionally been responsible for its own domestic policies on a variety of health issues, but given the cross-border, global nature of AMR, the onus has now become a collective one. To enact sustainable solutions on a global scale, countries with the highest level of influence must take the lead and advocate for united global action.

CC0 public domain photo by Wokandapix

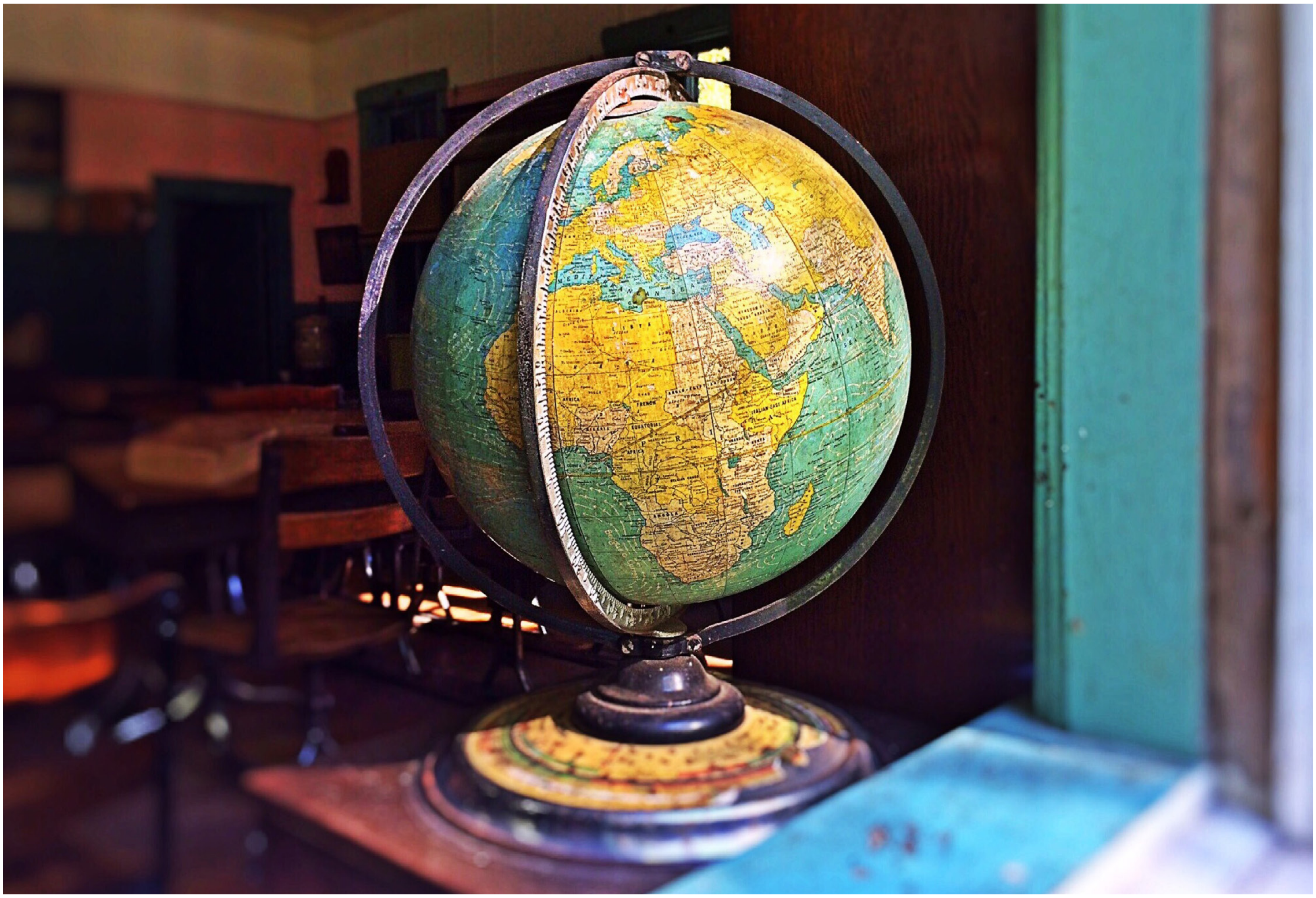

CONCLUSION

AMR is a naturally occurring phenomenon that cannot be naturally reversed. Combating AMR requires a coordinated and sustainable global response. Although many countries do not currently have the resources to take action, mobilizing core groups of Initiator and Pivotal Countries to take action on central issues can drive international commitments to access, conservation, and innovation measures. Initial efforts among a core group of countries could build the foundation that is necessary to develop a truly global and sustainable response. Like a ripple effect, targeted measures could lead to much broader action. This phased-in approach is, in all likelihood, the most politically feasible way to achieve global agreement on AMR.

Key Messages

1. The global AMR response must target access, conservation, and innovation challenges.
2. National governments, pharmaceutical companies, agriculture groups, civil society, and international organizations will be important stakeholders who must be engaged.
3. Many barriers to an international AMR agreement are likely to arise at different stages of the policy cycle, including agenda setting, agreement formulation, agreement legitimation, and agreement implementation.
4. For the purpose of assessing political feasibility of such an agreement, countries can be categorized into four types based on their global influence and potential interest in addressing AMR.
5. The political feasibility of an international agreement on AMR will depend on engaging a few key influential countries to develop policies that can eventually gain global traction.

REFERENCES

1. World Health Organization (WHO). WHO Website, Media Centre [Internet]. Geneva: WHO; 2015 Apr [updated 2015 Apr; cited 2015 Nov 15]. Available from: http://www.who.int/mediacentre/factsheets/fs194/en/

2. Hoffman SJ, Outterson K. Introduction: What Will It Take to Address the Global Threat of Antibiotic Resistance? J Law Med Ethics. 2015 Summer;43(2):6-11.

3. Hoffman SJ, Caleo GM, Daulaire N, Elbe S, Matsoso P, Mossialos E, Rizvi Z, Røttingen J. Strategies for Achieving Global Collective Action on Antimicrobial Resistance. Bull World Health Organ. 2015 Nov 19. Available from: http://www.who.int/bulletin/online_first/BLT.15.153171.pdf

4. Reich MR. The Politics of Reforming Health Policies. IUHPE - Prom & Ed. 2002;9(4):138-42.

5. Putnam RD, Diplomacy and Domestic Politics: The Logic of Two-Level Games. Int Organ. 1988;42(3):427-460.

6. Public Health Agency of Canada. Antimicrobial Resistance - A Shared Responsibility [Internet]. Canada: Public Health Agency of Canada; 2013 [cited 2015 Nov 15]. The Chief Public Health Officer's Report on the State of Public Health in Canada 2013 Infectious Disease - The Never Ending Threat. Available from: http://www.phac-aspc.gc.ca/cphorsphc-respcacsp/2013/resistance-eng.php

7. International Federation of Pharmaceutical Manufacturers & Associations (IFPMA). Rethinking the Way We Fight Bacteria. Geneva: IFPMA; 2015 [cited 2015 Nov 15].

8. Barlam TF, Gupta K. Antibiotic Resistance Spreads Internationally Across Borders. J Law Med Ethics. 2015 Summer;43(2):12-16.

9. Review on Antimicrobial Resistance. Review on Antimicrobial Resistance Website [Internet]. UK: Review on AMR; 2014 [cited 2015 Nov 15]. Available from: http://amr-review.org/home

10. Hollis A, Maybarduk P. Antibiotic Resistance is a Tragedy of the Commons that Necessitates Global Cooperation. J Law Med Ethics. 2015 Summer;43(2):33-37.

11. Daulaire N, Bang Abhay, Tomson G, et al. Universal Access to Effective Antibiotics is Essential for Tackling Antibiotic Resistance. J Law Med Ethics. 2015 Summer;43(2):17-21.

12. World Bank. GDP Ranking[Internet]. World Bank; 2014 [Updated 2016 Feb 17, Cited 2016 Feb 20]. Available from: http://data.worldbank.org/data-catalog/GDP-ranking-table

13. The Centre for Disease Dynamics, Economics & Policy (CDDEP) [Internet]. Methodology: Antibiotic Use. Available from: http://resistancemap.cddep.org/resmap/methodology/use

14. Santa-Ana-Tellez Y, Mantel-Teeuwisse AK, Dreser A, Leufkens HGM, Wirtz VJ. Impact of Over-the-Counter Restrictions on Antibiotic Consumption in Brazil and Mexico. PLoS One. 2013 Oct;8(10):1-6.

15. World Health Assembly Journal. Sixty-eighth World Health Assembly Statements - Republic of Korea [Internet]. 2015 May. Available from: http://apps.who.int/gb/Statements/WHA68/PDF/Committee%20A/Seventh%20meeting/REP%20OF%20 KOREA%20-%20AMR_ROK.pdf

16. Timmel C. AMR: Ambitious Initiatives of the US Government [Internet]. Semmelweis; 2016. Available from: http://semmelweis.info/4068-2/

17. White H. Working with Korea to Tackle Antimicrobial Resistance [Internet]. UK Foreign & Commonwealth Office; 2016. Available from: http://blogs.fco.gov.uk/hollywhite/2016/02/04/working-with-korea-to-tackle-antimicrobial-resistance/

18. The White House. Fact Sheet: President's 2016 Budget Proposes Historic Investment to Combat Antibiotic-Resistant Bacteria to Protect Public Health [Internet]. The White House Office of the Press Secretary; 2015. Available from: https://www.whitehouse.gov/the-press-office/2015/01/27/fact-sheet-president-s-2016-budget-proposes-historic-investment-combat-a

19. The White House. National Strategy for Combating Antibiotic-Resistant Bacteria [Internet]. The White House; 2014. Available from: https://www.whitehouse.gov/sites/default/files/docs/carb_national_strategy.pdf

20. US Food and Drug Administration. FDA's Strategy on Antimicrobial Resistance - Questions and Answers [Internet]. Maryland: FDA; 2015 Nov 6. Available from: http://www.fda.gov/AnimalVeterinary/GuidanceComplianceEnforcement/GuidanceforIndustry/ucm216939.htm#question9

21. Sifferlin A. FDA to Farmers: No More Antibiotics to Fatten Up Livestock [Internet]. Time. 2013 Dec 11. Available from: http://healthland.time.com/2013/12/11/fda-to-farmers-no-more-antibiotics-to-fatten-up-livestock/

22. US Food and Drug Administration. FDA Update on Animal Pharmaceutical Industry Response to Guidance #213 [Internet]. 2015. Available from: http://www.fda.gov/AnimalVeterinary/SafetyHealth/AntimicrobialResistance/JudiciousUseofAntimicrobials/ucm390738.htm

23. Maron DF, Smith TJS, Nachman KE. Restrictions on Antimicrobial Use in Food Animal Production: An International Regulatory and Economic Survey. Global Health [Internet]. 2013 Aug;48(9):1-11. Available from: http://globalizationandhealth.biomedcentral.com/articles/10.1186/1744-8603-9-48

24. Briones RM, Rakotoarisoa MA. FAO Commodity and Trade Policy Research Working Paper No. 38. Investigating the Structures of Agricultural Trade Industry in Developing Countries. Food and Agriculture Organization of the United Nations; 2013.

25. Centres for Disease Control and Prevention [Internet]. Protecting the Power of Antibiotics: Lessons from Egypt; 2015. Available from: http://www.cdc.gov/globalhealth/stories/protecting_power_antibiotics.htm

26. World Health Organization [Internet]. WHO Report Finds Systems to Combat Antibiotic Resistance Lacking. WHO Media Centre; 2015. Available from: http://www.who.int/mediacentre/news/releases/2015/antibiotic-resistance-lacking/en/

27. Sabry NA, Farid SF, Dawoud DM. Antibiotic Dispensing in Egyptian Community Pharmacies: An Observational Study. Res in Social Adm Pharm [Internet]. 2014 Jan-Feb;10(1):168-84. Available from: http://www.ncbi.nlm.nih.gov/pubmed/23665078

28. Abdulhak AAB, Tannir MAA, Almansor MA, Almohaya MS, Onazi AS, Marei MA, et al. Non Prescribed Sale of Antibiotics in Riyadh, Saudi Arabia: A Cross Sectional Study. BMC Public Health [Internet]. 2011 July;538(11):1-5. Available from: http://bmcpublichealth.biomedcentral.com/articles/10.1186/1471-2458-11-538

29. Al-Tawfiq JA, Mernish ZA. Potential Risk for Drug Resistance Globalization at the Hajj [Internet]. CMI. 2015 Feb;21(2):109-114. Available from: http://www.clinicalmicrobiologyandinfection.com/article/S1198-743X(14)00099-8/fulltext

30. Romanian Ministry of Health (Ministerul Sănătătii) [Internet]. Media Release (Comunicare de presă). 2016 Feb. Available from: http://www.ms.ro/?pag=62&id=15727&pg=1

31. Tomson G, Vlad I. The Need to Look at Antibiotic Resistance from a Health Systems Perspective. Upsala Journal of Medical Sciences [Internet]. 2014 May;119(2):117-24. Available from: http://www.ncbi.nlm.nih.gov/pubmed/24673267

32. Nguyen KV, Do NTT, Chandna A, Nguyen TV, Pham CV, Doan PM, et al. Antibiotic Use and Resistance in Emerging Economies: A Situation Analysis for Viet Nam. BMC Public Health [Internet]. 2013 Dec;13:1158. Available from: http://www.ncbi.nlm.nih.gov/pubmed/24325208

33. World Health Organization [Internet]. Viet Nam Signs Aide-memoire to Combat Antimicrobial Resistance. WHO Media Centre; 2015. Available from: http://www.wpro.who.int/mediacentre/releases/2015/20150722/en/

34. Cddep.org. Global Antibiotic Resistance Partnership [Internet]. 2015 [cited October 2015]. Available from: http://www.cddep.org/garp/home#sthash.vcxp8c1t.dpuf

35. Carrique-Mas JJ, Trung NV, Hoa NT, Mai HH, Thank TH, Campbell JI, et al. Antimicrobial Usage in Chicken Production in the Mekong Delta of Vietnam. Zoonoses and Public Health [Internet]. 2015 Apr;62(1):70-78. Available from: http://www.ncbi.nlm.nih.gov/pubmed/25430661

36. Appiah B. Africa Needs to Address Antimicrobial Resistance. SciDev.Net Sub-Saharan Africa [Internet]; 2015. Available from: http://www.scidev.net/sub-saharan-africa/health/editorials/africa-address-antimicrobial-resistance-health.html

37. Antibiotic Drug Use, Monitoring, and Evaluation of Resistance [Internet]. Communiqué - African Conference on Antibiotic Use and Resistance; 2015. Available from: http://admerproject.org/images/gimpa2015/CommuniqueAFCAR2015.pdf

38. Dimina E, Kula M, Caune U, Liepins M, Zeidaka L, Nikitina O, et al. Repeated Prevalence Studies on Antibiotic Use in Latvia, 2003-2007. Eurosurveillance [Internet]. 2009 Aug;14(33):1-5. Available from: http://www.ncbi.nlm.nih.gov/pubmed/19712639

39. European Centre for Disease Prevention and Control [Internet]. Country Mission Latvia: Antimicrobial Resistance; 2011. Available from: http://ecdc.europa.eu/en/publications/Publications/antimicrobial-resistance-latvia-country-mission.pdf

40. International Federation of Pharmaceutical Manufacturers & Associations (IFPMA). IFPMA Position on Antimicrobial Resistance (AMR). News Release [Internet]. 2011 Apr 7 [cited 2015 Nov 15]. Available from: http://www.ifpma.org/fileadmin/content/Innovation/Anti-Microbical%20Resistance/IFPMA_Position_on_Antimicrobial_Resistance_NewLogo2013.pdf

41. Pharmaceutical Research and Manufacturers of America (PhRMA). Economic Impact: The Biopharmaceutical Industry Helps Strengthen the U.S. Economy. PhRMA Website [Internet]. Washington: PhRMA; 2015 [cited 2015 Nov 15]. Available from: http://phrma.org/economic-impact

42. Spellberg B, Guidos R, Gilbert D, Bradley J, Boucher HW, Scheld WM, et al. The Epidemic of Antibiotic-Resistant Infections: A Call to Action for the Medical Community from the Infectious Diseases Society of America. Clin Infect Dis. 2008 Jan;46:155-64.

43. Review on Antimicrobial Resistance [Internet]. Declaration by the Pharmaceutical, Biotechnology, and Diagnostics Industries on Combatting Antimicrobial Resistance. 2016. Available from: http://amr-review.org/sites/default/files/Declaration_of_Support_for_Combating_AMR_Jan_2016.pdf

44. World Health Organization (WHO). The Medical Impact of the Use of Antimicrobials in Food Animals. Report of a WHO Meeting. Berlin, Germany; 13-17 Oct 1997.

45. Van Boeckel TP, Brower C, Gilbert M, Grenfell BT, Levin SA, Robinson TP, et al. Global Trends in Antimicrobial Use in Food Animals. PNAS. 2015;112(18):5649-54.

46. Landers TF, Cohen B, Wittum TE, Larson EL. A Review of Antibiotic Use in Food Animals: Perspective, Policy, and Potential. Public Health Reporters. 2012;127:4-22.

47. World Health Organization. Critically Important Antimicrobials for Human Medicine [Internet]. WHO Advisory Group on Integrated Surveillance of Antimicrobial Resistance; 2011. Available from: http://apps.who.int/iris/bitstream/10665/77376/1/9789241504485_eng.pdf

48. World Health Organization (WHO). Mobilizing Political Will to Contain Antimicrobial Resistance. Bull World Health Organ. 2011;89(3):168-69.

49. Casewell M, Friis C, Marco E, McMullin P, Phillips I. The European Ban on Growth-Promoting Antibiotics and Emerging Consequences for Human and Animal Health. J Antimicrob Chemother. 2003;52:159-61.

50. Animal Health Institute. The Antibiotic Ban in Denmark: A Case Study on Politically Driven Bans. 2015 [cited 2015 Nov 16]. Available from: http://www.ahi.org/issues-advocacy/animal-antibiotics/the-antibiotic-ban-in-denmark-a-case-study-on-politically-driven-bans/

51. Kesmodel D, Bunge J, McKay B. More Companies Go Antibiotics-free as More Consumers Demand It. The Wall Street Journal. 2014 Nov 3 [cited 2015 Nov 16]. http://www.wsj.com/articles/meat-companies-go-antibiotics-free-as-more-consumers-demand-it-1415071802

52. Abbott KW. Innovations in Global Health and the Global Governance System. In: Proceedings of the Wall Summer Institute for Research: Civil Society Organizations and Global Health Governance; 2007 June; Vancouver. Published online: SSRN; 2007. Available from: http://ssrn.com/abstract=1471362

53. Zacher MW, Keefe TJ. The Transformation of Global Health Governance: Utilization and Expansion of Control Strategies Since the 1990s. In: Proceedings of the Wall Summer Institute for Research: Civil Society Organizations and Global Health Governance; 2007 June 25; Vancouver. Published online: Research Gate; 2007.

54. Mutekanga D. Interview with David Mutekanga, GARP-Uganda Principal Investigator. 2015. Available from: http://www.cddep.org/blog/posts/interview_david_mutekanga_garp_uganda_principal_investigator

55. Faure K. Interview with Kim Faure, GARP-South Africa. 2015. Available from: http://www.cddep.org/blog/posts/interview_kim_faure_garp_south_africa

56. Wesangula E. Q&A with GARP-Kenya Coordinator Eveline Wesangula. 2015. Available from: http://www.cddep.org/blog/posts/qa_garp_kenya_coordinator_eveline_wesangula

57. Kentikelenis A, King L, McKee M, Stuckler D. The International Monetary Fund and the Ebola Outbreak. 2015 Dec 21;3(2):e69-e70.

58. Gostin LO. Reforming the World Health Organization after Ebola. JAMA. 2015 Apr 14;313(14):1407-08.

59. Bergstrom R. Why Pharma Has to Stop Playing Solo. Barcelona; 2014. Available from: http://www.pharmatelevision.com/VideoHandler.aspx?ID=1210

60. Townsend B, Martin E, Löfgren H, De Leeuw E. Global Health Governance: Framework Convention on Tobacco Control (FCTC), the Doha Declaration, and Democratisation. 2012 May 29 Administrative Sciences 2:186-202.

61. Weishaar H, Collin J, Smith K, Grüning T, Mandal S, Gilmore A. Global Health Governance and the Commercial Sector: A Documentary Analysis of Tobacco Company Strategies to Influence the WHO Framework Convention on Tobacco Control. June 2012 PLoS Med 9(6):e1001249.

62. World Health Organization. Resolution WHA 67.25: Antimicrobial Resistance. Geneva, World Health Assembly 67, 2014 [cited 2015 Oct 30]. Available from: http://apps.who.int/gb/ebwha/pdf_files/WHA67/A67_R25-en.pdf

63. Chioro A, Coll-Seck AM, Hoie B, et al. Antimicrobial Resistance: A Priority for Global Health Action. Bull World Health Organ [Internet]. 2015;93:439. [cited 2015 Nov 27] Available from: http://www.who.int/bulletin/volumes/93/7/15-158998.pdf

64. Fidler DP. Legal Issues Associated with Antimicrobial Drug Resistance. Centres for Disease Control and Prevention (CDC) [Internet]. 1998 June;4(2). Available from: http://wwwnc.cdc.gov/eid/article/4/2/98-0204_article

65. Wilson K, McDougall C, Upshur R. The New International Health Regulations and the Federalism Dilemma. PLOS Med 3(1) e1:30-34 [Internet]. 2006 Jan [cited 2015 Nov 21]; Available from: http://journals.plos.org/plosmedicine/article?id=10.1371/journal.pmed.0030001

66. World Health Organization (WHO). International Health Regulations (2005), 2nd ed, Appendix 2 Reservations and Other State Party Communications in Connection with the International Health Regulations (2005) [Internet]. Geneva: WHO; 2008 [cited 2015 Nov 21]. Available from: http://apps.who.int/iris/bitstream/10665/43883/1/9789241580410_eng.pdf

67. World Health Organization. Worldwide Country Situation Analysis: Response to Antimicrobial Resistance. Geneva. 2015 Apr 29.

68. World Health Organization (WHO). World Antibiotic Awareness Week [Internet]. Geneva: WHO; 2015 [cited 2015 Nov 21]. Available from: http://www.who.int/mediacentre/events/2015 /world-antibiotic-awareness-week/en/

69. World Health Organization (WHO). Antimicrobial Resistance - Global Report on Surveillance. Switzerland: WHO Press; 2014. Available from: http://apps.who.int/iris/bitstream/10665/112642/1/9789241564748_eng.pdf?ua=1

70. Gelband H, Miller-Petrie M, Pant S, et al. State of the World's Antibiotics [Internet]. Washington D.C.: Centre for Disease Dynamics, Economics and Policy; 2015 [cited 2015 Oct 30]. Available from: http://cddep.org/publications/state_worlds_antibiotics_2015

71. Grace D. Review of Evidence on Antimicrobial Resistance and Animal Agriculture in Developing Countries. International Livestock Research Institute. 2015.

72. Smith RD, Yago M, Millar M, Coast J. A Macroeconomic Approach to Evaluating Policies to Contain Antimicrobial Resistance. Appl Health Econ Health Policy. 2006;5(1):55-65.

73. Balasegaram M, Clift C, Røttingen J. The Global Innovation Model for Antibiotics Needs Reinvention. J Law Med Ethics. 2015 Summer;43(2):22-26.

74. GlaxoSmithKline. Global Public Policy Issues - GlaxoSmithKline's Position - Incentivizing Antibacterial R&D. GlaxoSmithKline Government Affairs, Public Policy and Patient Advocacy. 2012.

75. Daulaire N. Key Informant Interview. 2015 Oct 7 (Unpublished).

76. Behdinan A, Hoffman S J, Pearcey M. Some Global Policies for Antibiotic Resistance Depend on Legally Binding and Enforceable Commitments. 2015 Summer; 43(2): 68-73.

77. Cooper AF. Chapter 17, The G8/G20 and Global Health Governance: Extended Fragmentation or a New Hub of Coordination. In: Kickbusch I, Lister G, Told M, Drager N. Global Health Diplomacy. New York: Springer; 2013:243-52.

78. Kates J, Katz R. The Role of Treaties, Agreements, Conventions, and Other International Instruments in Global Health. Infect Dis Clin N Am. 2011;25(2):455-75.

79. Hoffman S J, Ottersen T. Addressing Antibiotic Resistance Requires Robust International Accountability Mechanisms. J Law Med Ethics. 2015 Summer; 43(2):53-64.

80. Abdula, N, Macharia J, Motsoaledi A, et al. National Action for Global Gains in Antimicrobial Resistance. The Lancet [Internet]. 2015 [cited 2015 Nov 18]. Available from: http://dx.doi.org/10.1016/S0140-6736(15)00668-6

81. Ratanawijitrasin S, Wondemagegnehu E. Effective Drug Regulation: A Multicountry Study. World Health Organization. 2002 [cited Nov 27 2015]. Available from: http://apps.who.int/medicinedocs/pdf/s2300e/s2300e.pdf

82. Laxminarayan R, Matsoso P, Brower C, et al. Access to effective antimicrobials: a worldwide challenge. The Lancet [Internet]. 2016;387:168 [cited 2016 March 8]. Available from: http://dx.doi.org/10.1016/S0140-6736(15)00474-2

83. Mendelson M, Røttingen JA, Gopinathan U, et al. Maximising access to achieve appropriate human antimicrobial use in low-income and middle-income countries. The Lancet [Internet]. 2016;387:188. Available from: http://dx.doi.org/10.1016/S0140-6736(15)00547-4

84. IMS Health [Internet]. Pharmerging Markets - Picking a Pathway to Success; 2012. Available from: https://www.imshealth.com/files/web/Global/Services/Services%20TL/IMS_Pharmerging_WP.pdf

85. OECD. Development aid stable in 2014 but flows to poorest countries still falling: Complete data tables. 2015 [cited 2016 Feb 5]. Available from: http://www.oecd.org/dac/stats/documentupload/ODA%202014%20Tables%20and%20Charts.pdf

86. Global Burden of Disease Study 2013. Global Burden of Disease Study 2013 (GBD 2013) Results by Location, Cause, and Risk Factor. Seattle, United States: Institute for Health Metrics and Evaluation (IHME), 2016.

87. IMS Health. Pharmerging Markets: Picking a Pathway to Success. White Paper. 2013. Available from: https://www.imshealth.com/files/web/Global/Services/Services%20TL/IMS_Pharmerging_WP.pdf

88. Dar OA, Hasan R, Schlundt J, et al. Exploring the evidence base for national and regional policy interventions to combat resistance. The Lancet [Internet]. 2016;387:285 [cited 2016 March 12].

89. Maiti R, Bhatia V, Padhy BM, et al. Essential Medicines: An Indian Perspective. The Indian Journal of Community Medicine [Internet]. 2015;40(4):223 [cited 2016 March 8]. Available from: http://www.ncbi.nlm.nih.gov/pmc/articles/PMC4581141/

90. Årdal C, Outterson K, Hoffman SJ, et al. International cooperation to improve access to access and sustain effectiveness of antimicrobials. The Lancet [Internet]. 2016;387:296 [cited 2016 March 12].

91. Danish Integrated Antimicrobial Resistance Monitoring and Research Programme.[Internet] Denmark:Statens Serum Institut, DTU Vet, DTU Food; [Updated 2015 Jul 17; cited 2016 Mar 19]. Available from: http://www.danmap.org/About%20Danmap.aspx

92. National Institute for Public Health and the Environment. NethMap 2015 [Internet]. 2015 June. Available from: http://www.swab.nl/swab/cms3.nsf/uploads/4F5A0D8E6F0DD139C1257E6E0051833A/$FILE/NethmapMaran2015%20_webversie.pdf

93. Norwegian Ministry of Health and Care Services. National Strategy Against Antibiotic Resistance 2015-2020. 2015. Available from: https://www.regjeringen.no/contentassets/5eaf66ac392143b3b2054aed90b85210/antibiotic-resistance-engelsk-lavopploslig-versjon-for-nett-10-09-15.pdf

94. HM Government (UK). UK 5 Year Antimicrobial Resistance (AMR) Strategy 2013-2018. 2014. Available from: https://www.gov.uk/government/uploads/system/uploads/attachment_data/file/385733/UK_AMR_annual_report.pdf

95. World Health Organization. Global Surveillance of Antibacterial Resistance in Humans 2nd WHO Technical Consultation[Internet]. 2014 March [cited 2016 March 20]. Available from:http://apps.who.int/medicinedocs/documents/s21640en/s21640en.pdf.

96. Dreser A, Vázquez-Vélez E, Treviño S, Wirtz VJ. Regulation of Antibiotic Sales in Mexico: An Analysis of Printed Media Coverage and Stakeholder Participation. BMC Public Health. 2012 Dec;12:1051. Available from: http://bmcpublichealth.biomedcentral.com/articles/10.1186/1471-2458-12-1051

97. Morgan DJ, Okeke IN, Laxminarayan R, Perencevich EN, Weisenberg S. Non-Prescription Antimicrobial Use Worldwide: A Systematic Review. Lancet Infect Dis [Internet]. 2011;11(9):692-701. Available from: http://www.ncbi.nlm.nih.gov/pmc/articles/PMC3543997/

98. Drug Office - Department of Health - The Government of Hong Kong Special Administrative Region [Internet]. Frequently Asked Questions. 2015 Dec 3. Available from: http://www.drugoffice.gov.hk/eps/do/en/pharmaceutical_trade/guidelines_forms/faq.html

99. Williams-Jones B, Béatrice D. Managing Antimicrobial Resistance in Food Production: Conflicts of Interest and Politics in the Development of Public Health Policy. Les ateliers de l'éthique. 2010;5(1):157-69.

100. Jeffries P. Key informant interview. 2016 Feb 23 (Unpublished).

101. Hughes P, Heritage J. Antibiotic-Growth Promoters in Food Animals. FAO [Internet]. 2002. Available from: http://www.fao.org/docrep/article/agrippa/555_en.htm

102. The College of Veterinarians of Ontario [Internet]. Use of Antimicrobial Pharmaceuticals in Food-Producing Animals: A Review. 2014 July. Available from:https://cvo.org/CVO/media/College-of-Veterinarians-of-Ontario/Resources%20and%20Publications/Reports/GF2BackgroundReview.pdf

103. European Medicines Agency [Internet]. Committee for Medicinal Products for Veterinary Use (CVMP) Strategy on Antimicrobials 2016-2020. 2015 Nov. Available from: http://www.ema.europa.eu/docs/en_GB/document_library/Scientific_guideline/2015/11/WC500196645.pdf

104. Laxminarayan M, Van Boeckel TP, Teillant A. The Economic Costs of Withdrawing Antimicrobials Growth Promoters from the Livestock Sector. OECD Food, Agriculture and Fisheries Papers. 2015;78:1-41.

105. Australian Veterinary Association and Animal Medicines Australia [Internet]. New Antibiotic Prescribing Guidelines for Livestock and Horses Will Help Preserve Effectiveness of Antimicrobial Medicines. Joint Media Release. 2015 Nov. Available from: http://www.ava.com.au/node/69942

106. Szkotnicki J. Key informant interview. 2015 Nov 6 (Unpublished).

107. Outterson K, Powers JH, Daniel GW, McClellan MB. Repairing the Broken Market for Antibiotic Innovation. Health Affairs [Internet]. 2015 Feb;34(2):277-285. Available from: http://content.healthaffairs.org/content/34/2/277.full.html

108. Deloitte Touche Tohmatsu. 2015 Global life sciences outlook, adapting in an era of transformation [Internet]. UK: Deloitte; 2014 [cited 2015 Nov 15]. Available from:
https://www2.deloitte.com/

109. International Federation of Pharmaceutical Manufacturers and Associations (IFPMA) [Internet]. The Pharmaceutical Industry and Global Health; 2015. Available from: http://www.ifpma.org/wp-content/uploads/2016/02/IFPMA_-_Facts_And_Figures_2015_web.pdf

110. The White House. National Action Plan for Combating Antibiotic-Resistant Bacteria [Internet]. Washington: The Administration; 2015. Available from: https://www.whitehouse.gov/sites/default/files/docs/national_action_plan_for_combating_antibotic-resistant_bacteria.pdf

111. Ostwald DA, Zubrzycki K, Knippel J. The Economic Footprint of the Pharmaceutical Industry - Regional Breakdown and Differentiation between Originators and Generics. WifOR [Internet]. 2015 Feb. Available from: http://www.ifpma.org/resource-centre/the-economic-footprint-of-the-pharmaceutical-industry/

112. PhRMA [Internet]. About PhRMA - PhRMA Members Leading the Way in Search of New Medicines and Cures. 2016. Available from: http://www.phrma.org/about

113. IMS Institute for Health Informatics [Internet]. Global Outlook for Medicines Through 2018 - Specialty Therapies Continue to Be More Significant in Developed Markets than in Pharmerging. 2014 Nov. Available from: http://static.correofarmaceutico.com/docs/2014/12/01/informe_ims.pdf

114. Webber DJ. Analyzing Political Feasibility: Political Scientists' Unique Contribution to Policy Analysis. Policy Stud J. 1986;14(4):545-53.

115. Meltsner AJ. Political Feasibility and Policy Analysis. Public Admin Rev. 1972;32(6):859-67.

116. Cairney P, Understanding Public Policy. Palgrave McMillan; 2012.

117. Kraft ME, Furlong SR, Public Policy: Politics, Analysis, and Alternatives 5th Edition. 2014.

APPENDIX

Follow the link below to view an additional web appendix ›
www.globalstrategylab.org/clinic/reports/feasibility-of-amr-agreement-2016_web-appendix.pdf

Acronyms

Term	Definition
› AGP	antibiotic growth promoter
› AMR	antimicrobial resistance
› BRICS	Brazil, Russia, India, China, and South Africa
› CDDEP	The Centre for Disease Dynamics, Economics & Policy
› CSOs	civil society organizations
› EU	European Union
› GARP	Global Antibiotic Resistance Partnership
› GDP	gross domestic product
› IHR	International Health Regulations
› LMIC	low- and middle-income countries
› MDR	multiple drug resistance/ multiple drug resistant
› OTC	over-the-counter
› R&D	Research and Development
› USA	United States of America
› WHO	World Health Organization

Methods

Stakeholder Analysis

We compiled the data for the stakeholder analysis through literature searching and key informant interviews. We reviewed published and grey literature to familiarize ourselves with AMR and to identify potential key informants. Discussions with key informants led in turn to new directions in literature searching. We carried out six thirty-minute semi-structured key informant interviews with stakeholders from governments, academia, industry, and non-governmental organizations. The interviews were used to guide our research and to explore the perspectives of these diverse groups of stakeholders. Semi-structured interviews were chosen to solicit information on the opinions and perceptions of key stakeholders. An initial interview guide was pilot tested during our first key-informant interview, and the guide was refined before other interviews were undertaken. The final interview guide comprised questions on international initiatives on AMR, the political feasibility of global agreement, and perspectives on access, innovation, and conservation. A note taker was present at each interview to capture the interviewee's responses.

Country Typology Analysis

To analyse political feasibility, we must consider how countries will act in the face of different proposed international agreements. Predicting how each country will act is a complex process: each country has its own considerations, history with AMR, and global standing.

We describe four simplified types of countries that might be engaged in an AMR agreement: Initiator Countries, Pivotal Countries, Follower Countries, and Neutral Countries. Categories were created on the basis of two factors: (1) global influence, and (2) interest in addressing AMR.

Global influence often depends on financial power. To categorize countries by global influence we used world ranking of GDP; the top 25% of countries by GDP were designated as the most influential.

Countries that have seen recent large increases in antibiotic consumption have a greater stake in combating AMR than countries with low or stable consumption rates. Yet, annual consumption rates, even standardized per capita, obscure underlying trends. For example, low rates of consumption might indicate either successful stewardship efforts or severe problems with antibiotic access. Evaluating annual rates might also be misleading due to short-term health and market shocks. To overcome these difficulties, we chose to report 10-year change in antibiotic usage as an indicator of interest in addressing AMR. We used 10-year percent change in human antibiotic consumption, based on data obtained from the Center for Disease Dynamics, Economics & Policy (CDDEP). The CDDEP data comprises information collected in the IMS Health MIDAS database, which uses antibiotics sold in retail and hospital pharmacies to estimate antibiotic consumption. Details on the methods of CDDEP can be found in citation 13. Individual data was available for 76 countries, while data for 6 countries in Central America and 10 countries in French West Africa were reported as a group. We collapsed countries into six categories based on the level of increase or decrease in antibiotic usage. The categories were: increased usage greater than 50%, 30-50% increase, 10-29% increase, neutral (less than 10% increase or decrease), 10 to 29% decrease, and 50% or greater decrease. For countries that CDDEP reported as a group we chose to assign the group antibiotic consumption level to each country, and to use their individual GDP rankings. This is not precise but provides an estimate sufficient for our purposes in defining the typology.

This measure gives a long term overview of AMR change at the national level. Whether the country had high or low consumption rates in 2000, a large proportional increase in consumption signals a need to re-examine national priorities, while a decrease indicates successful stewardship efforts. We plotted these GDP ranking and 10-year percent change in consumption against each other for 82 countries and developed the four types of countries illustrated in Figure 2 of our report. Data labels use the three letter notations from the World Bank; a full listing of where the 82 countries under consideration fall is presented in Figure A1.

Descriptions of the four types are included in the text of this report.

Our typology provides an organizing structure that assists in discussing the similarities of groups of countries. It was not possible to obtain newer data for a large number of countries and we recognize that some countries have made substantial progress more recently. We were also unable to capture animal use of antibiotics as this data is largely unavailable. We incorporate discussions of agricultural antibiotic use into our feasibility analysis to address this aspect of AMR.

Political Feasibility Analysis

Political feasibility has been described as an examination of actors and events in each stage of a policymaking process, and an anticipation of the likely resolution of the policy problem.[114] In defining political feasibility, Webber also outlined the goal of this type of analysis, which "… needs to assess the relative likelihood that a policy proposal or alternative, and a variety of modifications to that alternative, could be adopted and implemented in such a way that the policy problem is solved."[114]

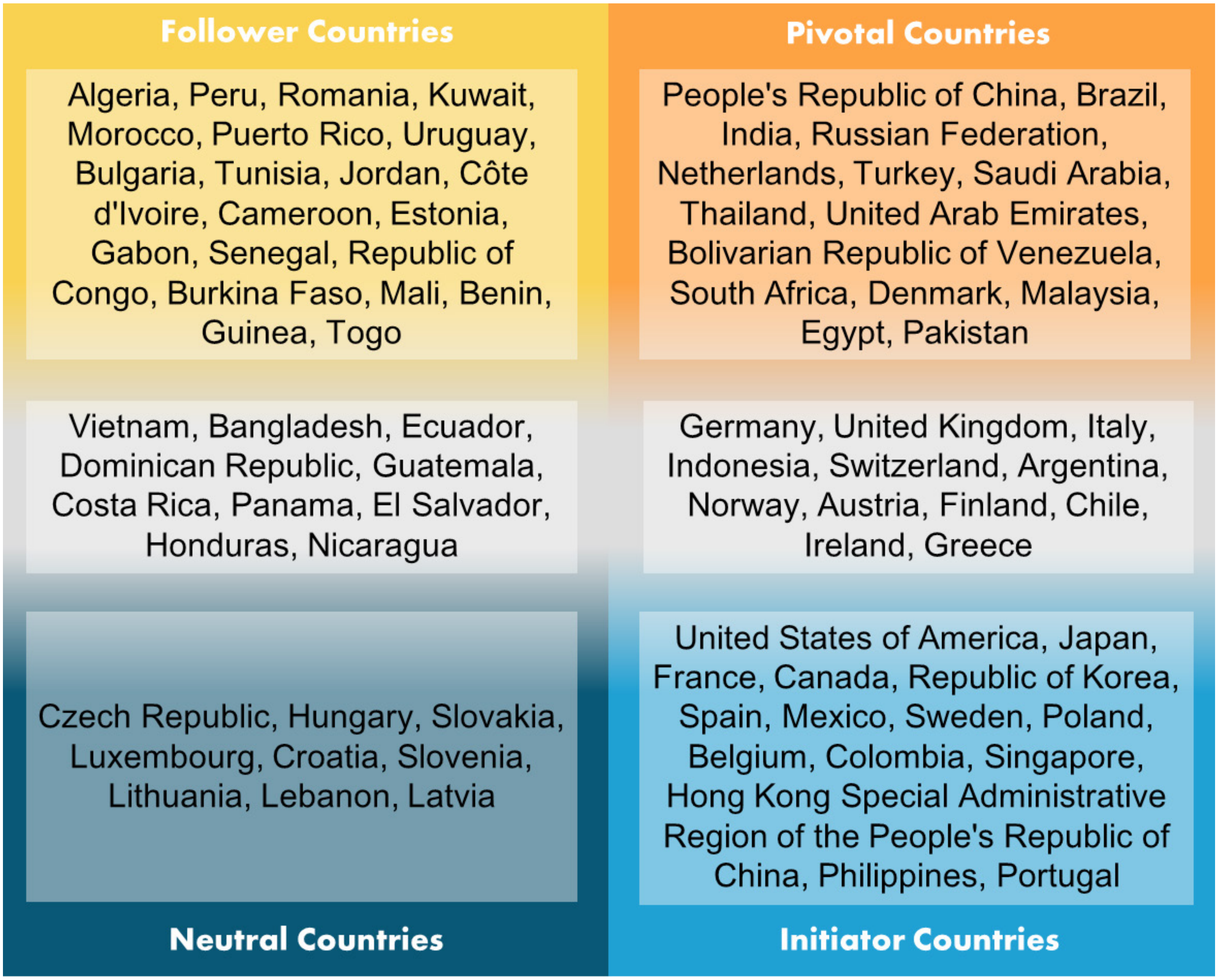

Figure A1: Full listing of countries by type

Webber's staged approach is valuable because it breaks the policy process into stages that can be individually examined. However, his approach necessitates having a clearly defined policy proposal as the starting point for a feasibility analysis. We need to take a more generalist approach to our political feasibility analysis, as we do not have a draft global AMR agreement to use as a starting point. Meltsner has described a different strategy for political feasibility analysis, which is more amenable to our early-stage analysis. Under his system, the analyst identifies actors, their beliefs and motivations, resources, and the sites of their interaction.[115]

Our analysis draws on both Meltsner's and Webber's methodologies. We adopt Webber's staged approach to analysis, using the well-known policy-cycle heuristic (Figure A2). In Part I of our report we identify barriers to an international agreement on AMR and the stage of the policy cycle where those barriers are likely to be problematic. As per Meltsner's methodology, we also identify key actors, their beliefs and motivations (here called interests), and interactions with other stakeholders.

Our barriers analysis centres on the policy-cycle, which breaks the policymaking process into 6 stages. We have chosen to focus on the first four stages: agenda setting, policy formulation, policy legitimation, and policy implementation. We identified important barriers in our literature review and key informant interviews, and categorized the in barriers by policy stage at which they are most problematic.

To determine the feasibility of policy proposals in Access, Innovation and Conservation, we considered the interlinkages between government priorities and capabilities, global AMR control needs, barriers to policy making, and stakeholder interests. On the basis of these factors we considered the likelihood that agreement on this issue could be achieved. Our analysis centres on rational choice theory: governments are expected to act on AMR in accordance with the best interests of their nation on matters of health and economics.

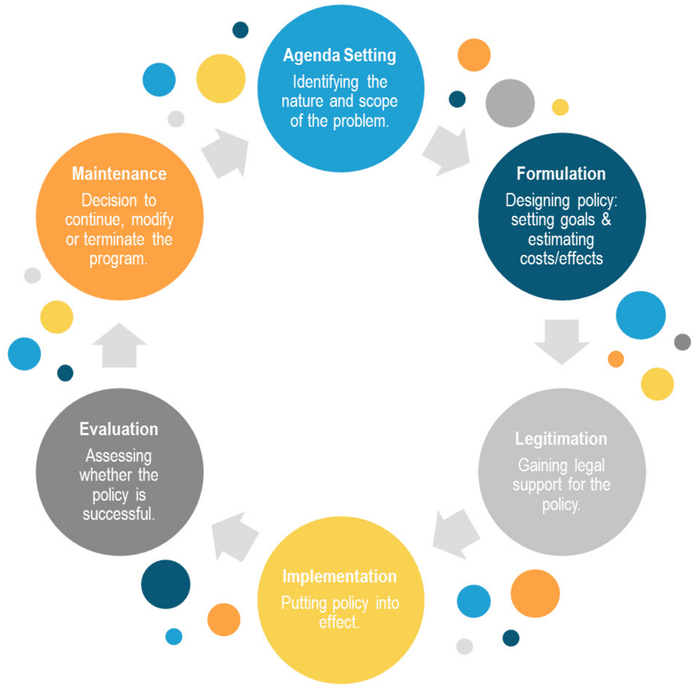

Figure A2. The Policy Cycle, adapted from Cairney[116] and Kraft[117]

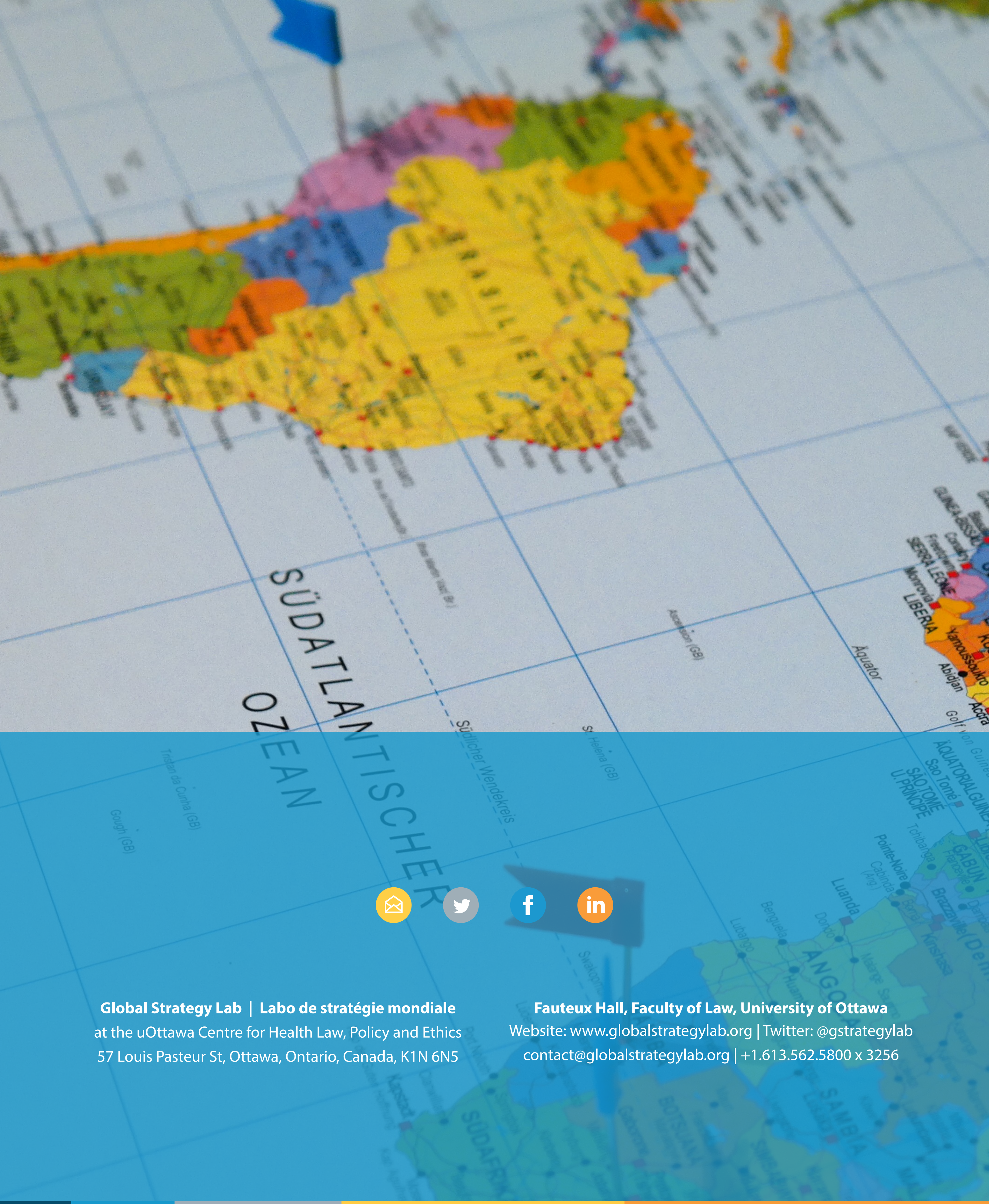

Global Strategy Lab | Labo de stratégie mondiale
at the uOttawa Centre for Health Law, Policy and Ethics
57 Louis Pasteur St, Ottawa, Ontario, Canada, K1N 6N5

Fauteux Hall, Faculty of Law, University of Ottawa
Website: www.globalstrategylab.org | Twitter: @gstrategylab
contact@globalstrategylab.org | +1.613.562.5800 x 3256
